# Cellular and molecular mechanisms of the blood–brain barrier dysfunction in neurodegenerative diseases

**DOI:** 10.1186/s12987-024-00557-1

**Published:** 2024-07-19

**Authors:** Tongli Chen, Yan Dai, Chenghao Hu, Zihao Lin, Shengzhe Wang, Jing Yang, Linghui Zeng, Shanshan Li, Weiyun Li

**Affiliations:** 1 School of Medicine, Hangzhou City University, Hangzhou, China; 2 Key Laboratory of Novel Targets and Drug Study for Neural Repair of Zhejiang Province, School of Medicine, Hangzhou City University, Hangzhou, China; 3 Institute of Brain and Cognitive Science, Hangzhou City University, Hangzhou, China

**Keywords:** Blood–brain barrier, Cerebrovascular blood flow, Vascular inflammation, Neurodegenerative diseases, Therapeutics

## Abstract

**Background:**

Maintaining the structural and functional integrity of the blood–brain barrier (BBB) is vital for neuronal equilibrium and optimal brain function. Disruptions to BBB performance are implicated in the pathology of neurodegenerative diseases.

**Main body:**

Early indicators of multiple neurodegenerative disorders in humans and animal models include impaired BBB stability, regional cerebral blood flow shortfalls, and vascular inflammation associated with BBB dysfunction. Understanding the cellular and molecular mechanisms of BBB dysfunction in brain disorders is crucial for elucidating the sustenance of neural computations under pathological conditions and for developing treatments for these diseases. This paper initially explores the cellular and molecular definition of the BBB, along with the signaling pathways regulating BBB stability, cerebral blood flow, and vascular inflammation. Subsequently, we review current insights into BBB dynamics in Alzheimer’s disease, Parkinson's disease, amyotrophic lateral sclerosis, and multiple sclerosis. The paper concludes by proposing a unified mechanism whereby BBB dysfunction contributes to neurodegenerative disorders, highlights potential BBB-focused therapeutic strategies and targets, and outlines lessons learned and future research directions.

**Conclusions:**

BBB breakdown significantly impacts the development and progression of neurodegenerative diseases, and unraveling the cellular and molecular mechanisms underlying BBB dysfunction is vital to elucidate how neural computations are sustained under pathological conditions and to devise therapeutic approaches.

## Background

**Fig. 1:**
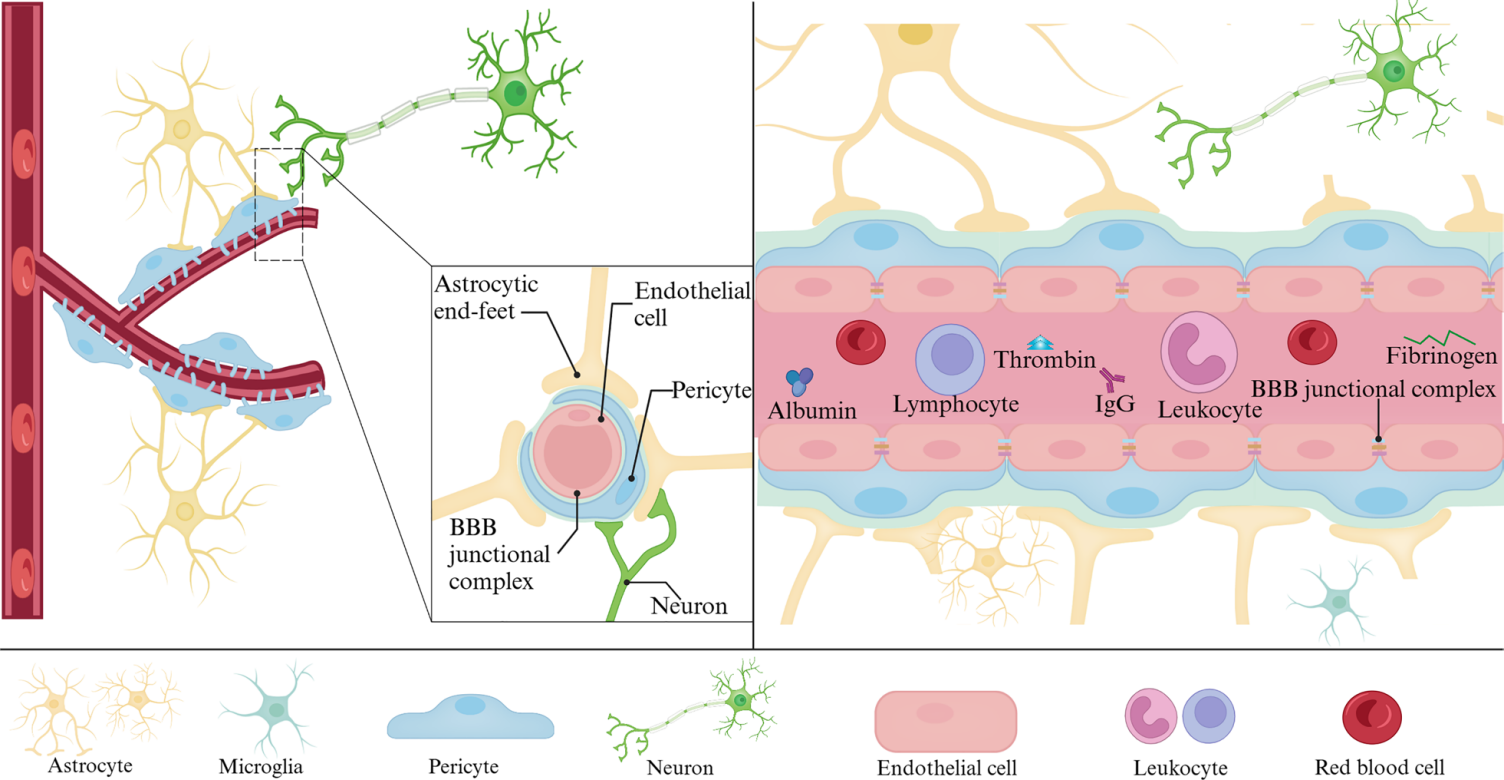
The NVU (capillary level). The NVU at the capillary level consists of vascular cells (ECs, pericytes, glial cells) and neurons. ECs, situated on the luminal side of blood vessels, are continuously sealed by intercellular junctional molecules like ZO proteins and claudins. The abluminal surface of ECs is enveloped by a basement membrane that incorporates pericytes and their projections. Astrocytic processes culminate in end-feet encircling the abluminal surface of the capillary vessel wall. The BBB functions to preserve neuronal health by preventing the unregulated influx of peripheral cells (such as red blood cells, leukocytes, and lymphocytes) and plasma proteins (e.g., fibrinogen, IgG). It also maintains low paracellular and transcellular permeability for molecules and ions while facilitating the delivery of essential oxygen and nutrients to the brain. Created with bioRender.com

The blood–brain barrier (BBB) serves as a crucial physical barrier separating peripheral blood circulation from the central nervous system (CNS). It safeguards the brain parenchyma by preventing the entry of exogenous pathogens and neurotoxic plasma components. Additionally, the BBB regulates substance transport into and out of the CNS, ensuring the chemical stability of the neuronal milieu and supporting normal neuronal metabolism, which is vital for the optimal functioning of the brain's 86 billion neurons. Impairments to the BBB disrupt the brain's microenvironment homeostasis, ultimately resulting in neuronal injury and functional anomalies, a phenomenon extensively documented in neurodegenerative disease studies [1]. The establishment and preservation of the BBB are intricate cooperative processes, hinging on the synergistic actions among the constituent cells of the neurovascular unit (NVU)—endothelial cells (ECs), pericytes, astrocytes, extracellular matrix, and neurons (Fig. 1). These cells, through complex interactions, guarantee the BBB’s integrity and the normal metabolic state of neurons [2, 3].

To uphold the BBB's integrity and support healthy neuronal metabolism, a continuous and sufficient blood supply is essential for the proper functioning of the brain. Adequate blood supply relies on an efficiently regulated vasculature and the NVU. ECs, pericytes, astrocytes, and neurons, among other cell types, collectively modulate cerebral blood flow (CBF) to cater to the high-energy demands of highly active brain regions [2, 3]. Impaired BBB integrity, such as the loss or degeneration of pericytes, disturbs regional CBF, disrupting the delicate balance required for optimal neural function [4–6]. Conversely, abnormal CBF also stresses the BBB, potentially contributing to its dysfunction and exacerbating the challenges faced by the vasculature. This intricate interplay between blood supply and brain health underscores the dual nature of the circulatory system's task: while it serves as a vital conduit for nourishment, it simultaneously manages the influx of potential toxins and mitigates the effects of oxidative stress. The process of nourishing the brain through the peripheral bloodstream inevitably involves the transport of not only essential nutrients but also potentially harmful elements. Simultaneously, the vasculature is confronted with managing oxidative stress products originating from both the peripheral circulation and the metabolic processes within the brain tissue. When confronted with harmful substances such as toxins or byproducts of oxidative stress in the periphery or brain tissue, the vascular system flexibly initiates defensive mechanisms to mitigate potential harm, demonstrating an adaptive response that protects the BBB’s protective function. However, excessive or persistent vascular inflammatory response may cause damage to blood vessels and surrounding tissues, affecting the BBB integrity and thus adversely affecting brain function. This involves immediate responses and refined coordination among various parts of the vasculature, underpinned by intricate signaling pathways and intercellular communication [7, 8]. The BBB's regulation exhibits a high sensitivity and responsiveness to energy requirements and environmental changes. The role of BBB in the pathogenesis of human neurodegenerative disorders highlights the importance of healthy blood vessels [9, 10]. Existing studies on neurodegenerative diseases reveal impairments related to BBB dysfunction, including compromised vascular stability, disrupted CBF, and vascular inflammation. However, in-depth investigations into the pathology of neurodegenerative diseases from the three perspectives, despite being crucial aspects, remain limited. There is an urgent need for a comprehensive review of the mechanisms of the BBB impairment, especially when considering the trio of crucial aspects in the context of various neurodegenerative conditions.

In this review, we initially explore the cellular and molecular mechanisms that underpin the formation of the BBB. Subsequently, our focus shifts to the pivotal pathways that maintain BBB integrity and regulate CBF, as well as vascular inflammation. Further, we investigate the connection between BBB dysfunction and neurodegenerative diseases, predominantly in Alzheimer’s Disease (AD), Parkinson’s Disease (PD), amyotrophic lateral sclerosis (ALS), and multiple sclerosis (MS). Finally, we examine potential BBB-based therapeutic strategies and targets. The review concludes by summarizing key insights and future research directions.

## The NVU

### EC

Specialized brain ECs construct the primary barrier interface of the BBB, maintaining physiological homeostasis by overseeing transport logistics, regulating vascular permeability, and controlling vascular tone [11]. In contrast to the highly permeable vasculature in peripheral organs, adjacent ECs in the brain are characterized by high trans-endothelial electrical resistance (TEER), low rates of transcytosis, and restricted paracellular permeability, which is primarily governed by junctional molecules [12]. The brain junctional molecules include tight junctions (TJs), adherens junctions (AJs), and gap junctions, which collectively impede the paracellular movement of solutes [13–16]. TJs, composed of claudins, occludin, junctional adhesion molecules (JAMs), and the zonula occludin (ZO) family, are believed to regulate vascular paracellular transport by forming a high-resistance electrical barrier [17]. Adherens junctions, primarily made up of vascular endothelial (VE)-cadherin, catenin, and platelet endothelial cell adhesion molecule-1 (PECAM-1), are located on the basolateral side of EC TJs [18]. They play a crucial role in trans-endothelial migration of lymphocytes, monocytes, and neutrophils, as well as in cell signaling and transcriptional regulation [19–21]. Gap junctions facilitate intercellular communication among ECs through ion currents, second messenger signals, and small metabolites. They are instrumental in vascular angiogenesis and modulating brain endothelial barrier hyperpermeability by influencing TJs. Nonetheless, a more comprehensive understanding of gap junctions concerning the BBB in neurodegenerative diseases is still required.

#### Transcellular permeability

Given the integrity of cellular structures and junctional proteins between ECs, it is typical for gases and small lipid-soluble molecules with molecular weights under 400 Da to be able to passively diffuse across the BBB [22]. The BBB employs various transport systems, such as carrier-mediated transport (CMT), receptor-mediated transport (RMT), active efflux transport, vesicular trafficking by transcytosis, and ion transport, to facilitate the entry of essential substances into the brain parenchyma and the efflux of metabolic waste and endogenous neurotoxins [19, 23]. The endothelial solute CMT facilitates the crossing of specific solutes across the BBB via substrate-specific transporters, with docosahexaenoic acid (DHA) being transported by major facilitator superfamily domain containing 2a (Mfsd2a) and glucose by glucose transporter 1 (Glut1), ensuring targeted delivery of these vital nutrients to the brain [19, 24–27]. RMT allows protein movement in and out of the brain, depending on the ligand's binding to specific plasma membrane receptors, such as the interaction between lipoprotein receptors 1 and 2 (LRP1/2) and advanced glycation end products (RAGE) [28, 29]. The ATP-binding cassette (ABC) transporters, including P-glycoprotein (P-gp) and breast cancer resistance protein (BCRP), expressed on the luminal endothelial plasma membrane, are pivotal in CNS pharmaco-resistance [24, 30–32]. Vesicular trafficking by transcytosis, exemplified by albumin's caveolae-mediated vesicular transport, allows molecules to traverse the BBB based on charged interactions with the ECs' glycocalyx [33]. Ion transporters, such as sodium pumps, calcium transporters, and potassium channels, are essential in regulating neuronal cell electrophysiological activity and maintaining CNS ion concentration gradients [24].

#### Leukocyte adhesion molecules

As vital constituents of the immune system, ECs act as conduits for migrating immune cells. Notably, ECs display considerably reduced levels of intercellular adhesion molecules (ICAMs) in comparison to ECs in peripheral organs, thereby imposing stringent limitations on immune cell ingress into the brain tissue [34, 35]. The traversal of immune cells from the circulation across the BBB is a meticulously orchestrated dance of molecular engagement between leukocytes and ECs. This intricate process, applicable to monocytes and neutrophils, includes initial capture and rolling along the EC lining, followed by integrin activation, adhesion, crawling, and eventual passage either transcellularly or paracellularly [36].

Under inflammatory cues, ECs instigate the expression of P-selectin, causing incoming immune cells to decelerate and roll. Under the orchestration of integrins and their ligands ICAM and VCAM, these cells firmly anchor to the EC surface, undergo polarization, and crawl in preparation for crossing. They then navigate across the endothelial layer either by traversing the TJs or by forming transient pores in the endothelium without compromising the structural integrity of complex TJs. Importantly, monocytes possess the capability to stimulate ECs directly, which may lead to decreased occludin levels and a heightened influx of immune cells into the inflamed site [27].

The leukocyte adhesion molecules located on the apical surface of ECs play a pivotal role in initiating leukocyte attachment, marking the onset of tissue infiltration. In a healthy state, ECs in the CNS exhibit significantly reduced expression of these molecules compared to their peripheral counterparts, further tightly controlling the infiltration of immune cells entry into the brain tissue [34, 35]. Conversely, in pathological conditions, an upsurge in leukocyte adhesion molecule expression on ECs eases the passage of leukocytes across the BBB, culminating in a local buildup of immune cells and intensifying both inflammatory and immune reactions. This amplification exacerbates harm to neurons [37, 38].

### Pericyte

Pericytes occupy a central position in the NVU, situated between ECs, astrocytes, and neurons [39]. Embedded in the basement membrane (BM), they are distinguished by their protruding ovoid cell bodies and elongated, slender processes that partly form an incomplete layer over the surface of CNS capillaries [40, 41]. Pericytes are instrumental in regulating several critical neurovascular functions. These include the formation and permeability of the BBB, angiogenesis, CBF, the clearance of toxic cellular metabolites, vascular remodeling, and tone, all of which are essential for the normal functioning of the CNS [39].

Pericytes, responsive to signals from neighboring cells, generate functional responses essential for CNS integrity [39]. During CNS development, pericyte-EC interactions regulate angiogenesis, vascular stability, and remodeling [42, 43]. Pericytes influence vascular permeability by modulating the expression and organization of intercellular junction proteins and vesicular transport [44]. They regulate capillary tone and diameter, thereby controlling CBF, a topic further elaborated in the subsequent section of this review. In multiple neurological disorders, including AD, ALS, and PD, pericyte loss and degeneration are evident [19, 45, 46]. Pericyte deficiency intensifies amyloid-beta (Aβ) and tau pathology and accelerates neuronal loss in the absence of Aβ-precursor protein, leading to cognitive decline [45]. Moreover, pericytes' enhanced expression of adhesion molecules, which guides and facilitates leukocyte activation and transmigration into the CNS, underscores their role in mediating inflammatory signals within the NVU [47]. Neuronal activity drives insulin-like growth factor 2 expression from pericytes to form long-term memory [48].

### Astrocyte

Perivascular astrocytic end-feet cover approximately 70–100% of the vascular perimeter [49]. About a third of astrocyte soma directly contacts blood vessels [50]. As integral components of the NVU, perivascular astrocytes maintain BBB characteristics by contributing to basement membrane formation, regulating pericyte differentiation, and modulating EC functions by regulating the expression of TJs such as claudin-5, occludin, and ZO-1 [49, 51–53]. Astrocytes, also serving as pivotal relay cells within the NVU, facilitate communication between neurons and cerebral blood vessels [54]. Beyond neurotransmitter-mediated signaling and pericyte involvement, astrocytes regulate CBF through mechanisms linked to increased intracellular Ca^2+^ levels [7, 54]. Shifts in understanding CBF control will be elaborated upon in the following section. Additionally, heterogeneous astrocytes distributed throughout the brain exacerbate disease progression and compromise the BBB by releasing inflammatory factors and chemokines [55, 56]. Aquaporin-4 (AQP4), predominantly expressed in astrocytic end-feet, regulates the flux of cerebrospinal fluid, clearance of amyloid-beta, and inflammation [57, 58]. In intramural periarterial drainage, astrocytic end-feet are hypothesized to facilitate waste clearance by modulating functional hyperemia and cerebrovascular tone [59, 60]. AQP4 deficiency in mice leads to severe neuroinflammation, increased microglial activity, and neuronal damage [61].

### Basement membrane

The BM encompasses the abluminal surface of ECs and pericytes, delineating these cells from neurons and glial cells [62, 63]. BM components form a meshwork that facilitates the diffusion and binding of molecules and serves as an interface for cell adhesion and migration [64]. Comprising a structural matrix of extracellular matrix molecules, including collagens, laminins, fibronectin, heparan sulfate, and proteoglycans, the BM is synthesized and deposited by ECs, astrocytes, and pericytes [41, 65]. Its composition is tissue-specific and varies in response to different neurological diseases. Emerging evidence from human and animal studies of neurological diseases indicates that BBB disruption is associated with changes in BM component synthesis and degradation [64]. In the CNS, laminin deficiency leads to BBB breakdown, characterized by reduced pericyte coverage, leukocyte extravasation, and altered expressions of VE-cadherin, claudin-5, and occludin, underscoring laminin's role in maintaining BBB integrity [51, 66–68]. Collagen IV is essential for BBB structural integrity and function during later developmental stages [69]. Perlecan, a prominent heparan sulfate proteoglycan in BM, is crucial in BBB maintenance and repair, particularly in pericyte interactions following brain injuries like ischemic strokes [70–72]. Further research into BM's role in neurological disorders is critically necessary.

### Pathways regulating the BBB stability

**Fig. 2:**
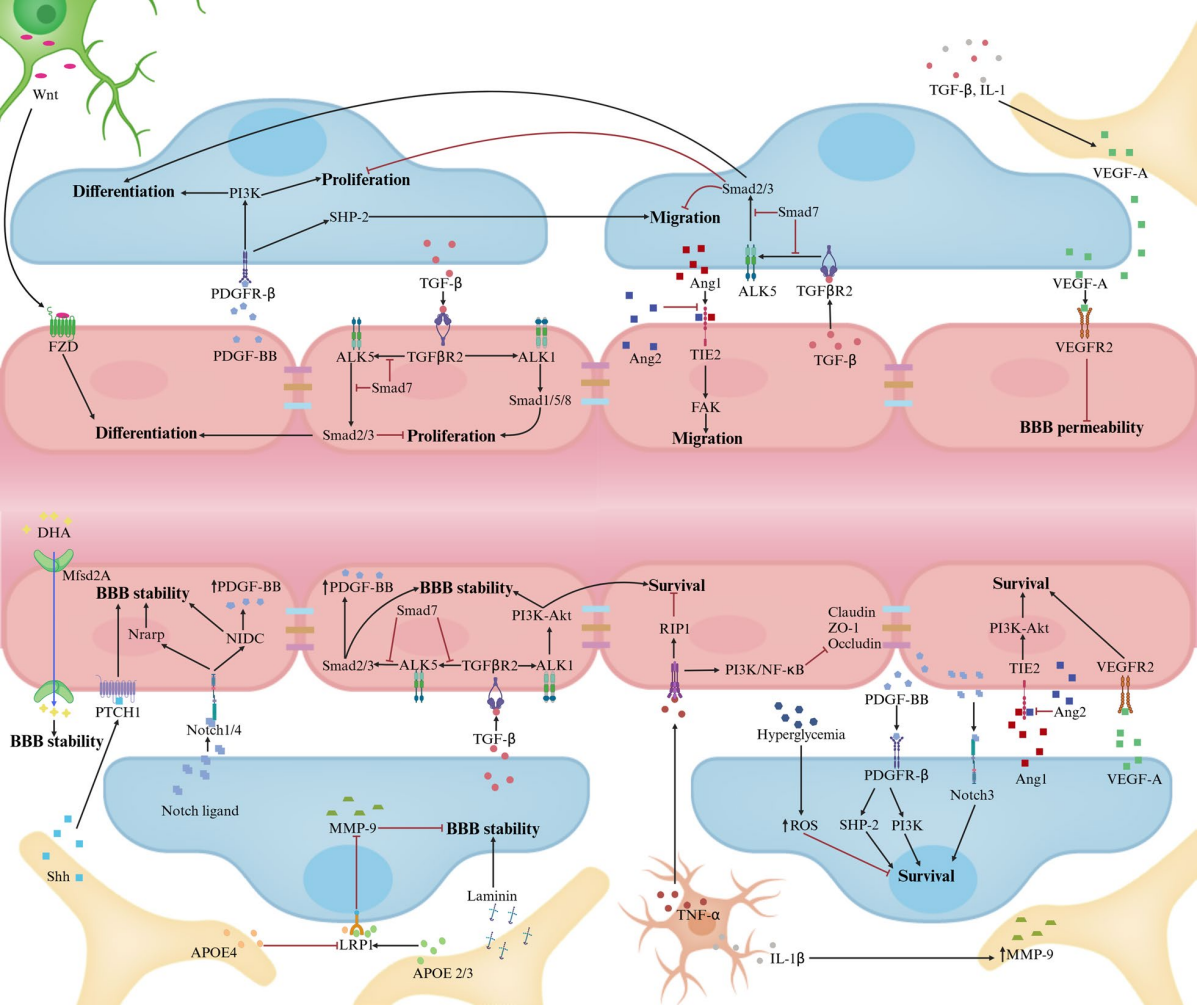
Major signaling pathways regulating BBB stability. Pericyte-EC communications. PDGF-BB-PDGFRβ pathways regulate various pericyte functions (survival, migration, proliferation, differentiation) by activating phosphoinositide 3-Kinase (PI3K) and Src homology-2 domain-containing protein tyrosine phosphatase-2 (SHP-2). In ECs, TGF-β–TGFβR2 activates different cascades: Alk5-Smad2/3/4 complex boosts differentiation, curbs proliferation, Alk1-Smad1/5/8 complex enhances proliferation; and Alk1-PI3K–Akt pathway fortifies survival and BBB stability. In pericytes, Smad2/3 activation restrains proliferation and migration, promoting differentiation. Notch3 receptor engagement by Notch ligands strengthens BBB stability through Notch-regulated ankyrin repeat protein (Nrarp) or Notch intracellular domain (NICD) pathways when binding to Notch1 or Notch4. Excessive VEGF-A from pericytes and astrocytes weakens BBB by interacting with EC's VEGFR2. Ang1 binding to Tie2 receptors activates PI3K–Akt and inhibits β-catenin, supporting BBB stability, whereas Ang2 antagonizes this effect. Astrocyte-Pericyte/ EC communications. Astrocytes secrete APOE2 and APOE3 (not APOE4), which bind to pericyte LRP1 receptors, inhibiting the CypA–NFκB–MMP-9 pathway and bolstering BBB stability. Moreover, astrocytic Shh binds to PTCH1 receptors on ECs, playing a critical role in BBB stabilization. Astrocyte-derived laminin significantly supports BBB integrity. Neuron-EC communications. Neurons release Wnt, which upon binding to frizzled (FZD) receptors on ECs stimulates their differentiation. Microglia-EC Communications. Microglia release TNF-α and IL-1β, which cumulatively diminish the stability of BBB. Other factors influencing BBB integrity. Additional factors include MFSD2A's role in BBB formation and hyperglycemia-induced pericyte apoptosis via ROS

The structural stability of the BBB is a prerequisite for its normal functional performance, and anomalies in its structure may impact its selective permeability, thereby influencing the stability of the brain’s internal environment and the health status of neurons. The stability of the BBB largely depends on its vascular integrity and selective permeability. Interactions among ECs, pericytes, and astrocytes are crucial for vascular maintenance. Interventions targeting the processes of survival, proliferation, migration, differentiation, and permeability of vascular components, as well as the signaling pathways governing these processes, all have significant impacts on the stability of BBB. Disruptions to these may result in structural abnormalities, vessel leakage, and BBB functional impairments, consequently affecting brain functioning. The regulation of BBB permeability by various transporters has been extensively discussed elsewhere [31]. Figure 2 provides a summary of the key cellular and molecular pathways underlying BBB stability.

Platelet-derived growth factor-BB (PDGF-BB), upon binding to its receptor PDGF receptor-β (PDGFRβ), triggers receptor dimerization, autophosphorylation, and activation [39]. The PDGF-BB–PDGFRβ pathway and its downstream signaling mechanisms regulate BBB maintenance by promoting pericyte survival, proliferation, migration, differentiation, adherence to the vascular wall, and leukocyte trafficking [42, 73]. Endothelial-derived impaired PDGFRβ signaling results in microvascular reductions, decreased CBF, and accumulation of neurotoxic molecules from the blood, contributing to secondary neurodegeneration [45, 74]. Endothelium-derived Notch ligands binding to pericyte Notch3 receptors promote pericyte survival. Pericyte-originated Notch ligands attaching to endothelial Notch1/4 receptors enhance N-cadherin expression and regulate PDGF-BB in ECs, reinforcing BBB integrity [39]. The vascular endothelial growth factor-A (VEGF-A)–VEGFR2 signaling cascade is critical for cell survival, angiogenesis, and vascular permeability [42, 75]. However, aberrant activation of this pathway disrupts TJs, causing increased BBB leakage in CNS diseases [76, 77]. The transforming growth factor-β (TGF-β)–TGF-β receptor 2 (TGFβR2) signaling pathway regulates differentiation, maturation, proliferation, migration, and attachment of both ECs and pericytes [78, 79]. The angiopoietin-1 (Ang1)–Tie2 and Ang2–Tie2 signaling pathways, involved in angiogenesis and BBB permeability regulation, have varying effects on EC functions.

Transporters in ECs play a pivotal role in maintaining BBB stability. For example, Mfsd2a, an essential omega-3 fatty acid transporter in ECs vital for BBB formation, maintains BBB integrity by suppressing caveolae-mediated transcytosis [80, 81]. The brain's uptake of glucose is facilitated by Glut1. Significantly, deletion of a single allele encoding Glut1 markedly reduces glucose uptake, leading to BBB disruption, a decline in CBF, and ultimately, neuronal death and loss [27].

Beyond the previously mentioned pathways, perivascular microglia and astrocytes are critical in sustaining BBB stability. These cells release cytokines that interact with the BBB. Proinflammatory cytokines compromise ECs, stimulate the release of ICAM, and enhance the adhesion and infiltration of immune cells. This process amplifies inflammatory responses, contributing to the onset of CNS disorders [82]. Moreover, activated microglia phagocytose astrocytic end-feet, lead to the loss of astrocyte-vascular connections and subsequent BBB disruption [83, 84].

### Pathways regulating the CBF

**Fig. 3:**
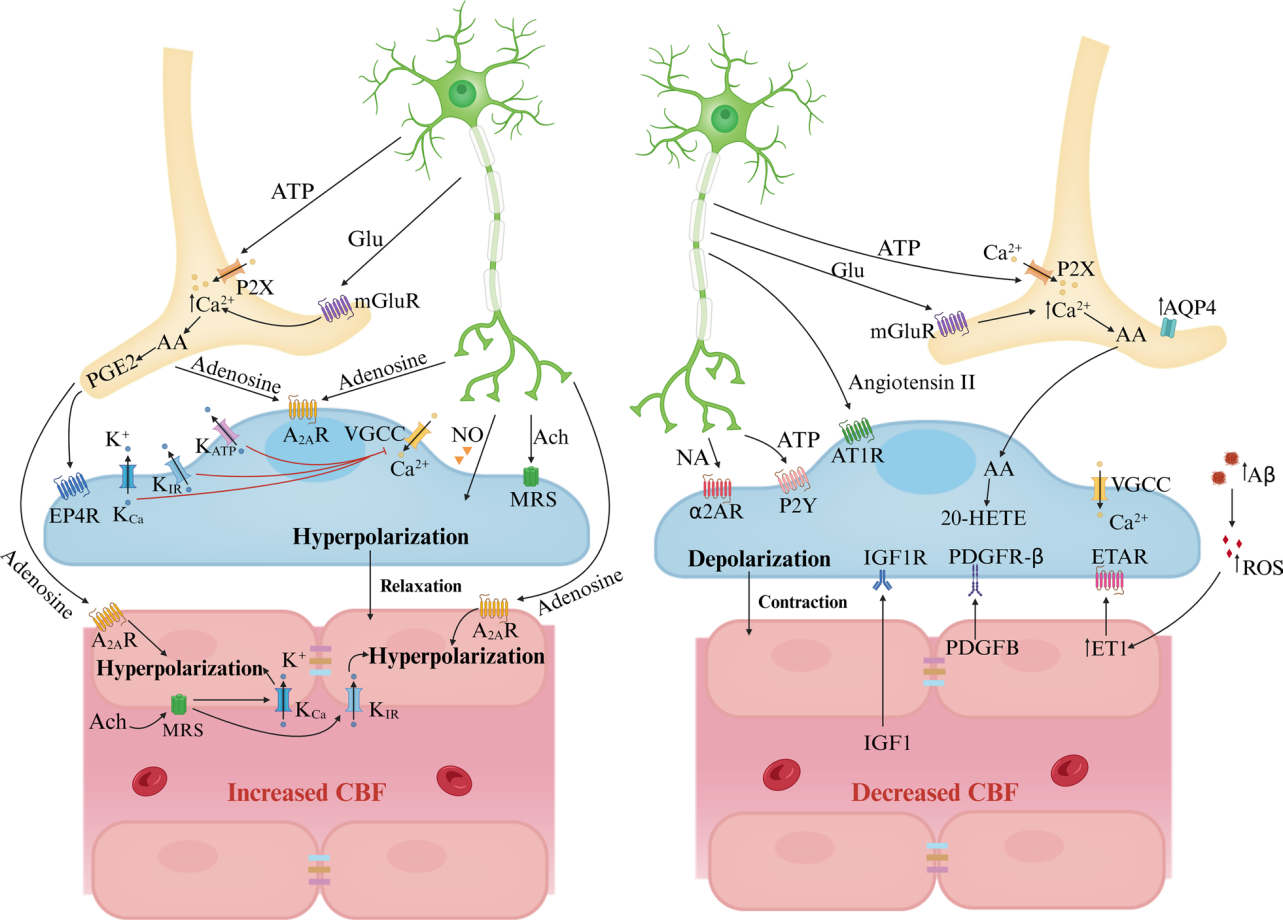
Major signaling pathways regulating CBF (capillary level). Increased CBF. Neuronal ATP and glutamate activate P2X receptors and metabotropic glutamate receptors (mGluRs) on astrocytes respectively, triggering Ca^2+^ influx. Elevated cytoplasmic Ca^2+^ stimulates COX-2 activity, leading to the conversion of AA into prostaglandin E2 (PGE2). PGE2 induces pericyte relaxation by binding to its receptor EP4 (EP4R), causing pericyte hyperpolarization. Similarly, acetylcholine released by neurons and adenosine from neurons and astrocytes target pericytes and ECs through muscarinic receptors and A_2A_Rs respectively, resulting in hyperpolarization and relaxation, thereby increasing CBF. Furthermore, high extracellular K^+^ from neurons activates VGCCs, causing pericyte depolarization and contraction. Conversely, activation of channels like the inward rectifier potassium channel and KCa or K_ATP_ channels leads to K^+^ efflux and pericyte hyperpolarization, reducing Ca^2+^ entry through VGCCs. ACh or high extracellular K^+^ can stimulate K_IR_ or K_Ca_ channels on ECs, causing endothelial hyperpolarization that propagates via gap junctions between ECs, enhancing CBF in a retrograde manner. Decreased CBF. Neurogenic ATP or NA binds to P2X or α2A receptors, respectively, on pericytes, resulting in cell depolarization and constriction, thereby reducing CBF. In astrocytes, AA is synthesized and then enters smooth muscle cells and pericytes where it transforms into 20-HETE, leading to pericyte depolarization, contraction, and a subsequent decrease in CBF. In AD models, excess Aβ triggers ROS generation in pericytes, upping ET-1 levels and inducing pericyte contraction and capillary constriction via ET-1 type A receptor (ETAR). Concurrently, EC-released PDGFB and blood-borne IGF1 interact with PDGFRβ and IGF1R, respectively, causing Ca^2+^ influx and pericyte depolarization. Moreover, brain pericyte AT1 receptors (AT1R) respond to neurogenic angiotensin II, amplifying contractile ability

The BBB’s primary role is to regulate the exchange of substances between blood and brain tissues, ensuring that essential nutrients reach neurons while excluding potential toxins and pathogens. CBF delivers the necessary oxygen and nutrients to the brain cells, and its regulation is dynamically adjusted according to the metabolic needs of the neurons. The integrity of the BBB is fundamental to this progress since it affects the vascular response and the overall efficiency of blood delivery. The human brain's blood supply is managed through a vascular network of arteries, arterioles, capillaries, and venules [26]. Given the predominance of capillaries in the brain's vasculature, their significant role in vascular resistance, and the extensive review of CBF control by smooth muscle cells (SMCs) elsewhere [54], this discussion focuses on capillary-based CBF regulation [26]. The NVU upholds BBB integrity and modulates CBF. Disruptions in the NVU, leading to BBB dysfunction and reduced CBF, are early events in various neurodegenerative diseases, including AD, PD, and ALS [85]. Indeed, the cellular components of the NVU play a crucial role in physiologically regulating CBF. This review explores how the brain modulates CBF in response to different information-processing tasks. Figure 3 presents the key cellular and molecular pathways involved in capillary-based CBF regulation.

#### Neuron-mediated regulation of CBF

Neurons release chemical signals that directly or indirectly influence receptors on pericytes and astrocytes, resulting in either decreased CBF due to depolarization and cell contraction or increased CBF through hyperpolarization and cell relaxation. Earlier studies posited that CBF is contingent on the energy demands of local neuronal activities. Following the generation of synaptic and action potentials, neurons use ATP released by glutamate to restore ion gradients, creating a metabolic signal that elevates CBF to meet energy requirements [86]. Recent research indicates that neuronal ATP and adenosine interact with their respective receptors-P2X and P2Y for ATP, and adenosine A_2A_ receptors (A_2A_R) for adenosine-leading to pericyte contraction, which reduces CBF, or membrane hyperpolarization, which increases CBF [26, 87–89]. Additionally, neuronal noradrenaline (NA) and angiotensin II act on α2-adrenergic receptors (α2A) for NA and AT1 for angiotensin II on pericytes. This action causes local Ca^2+^ elevations in pericytes, leading to depolarization and reduced CBF [90, 91]. Acetylcholine (ACh) released from neurons activates muscarinic ACh receptors (MRs) on ECs, increasing nitric oxide (NO) production, EC hyperpolarization, and consequently, elevated CBF [92]. NO released by neurons influences pericytes, causing hyperpolarization and exerting vasodilatory effects, further impacting CBF [93].

**Fig. 4:**
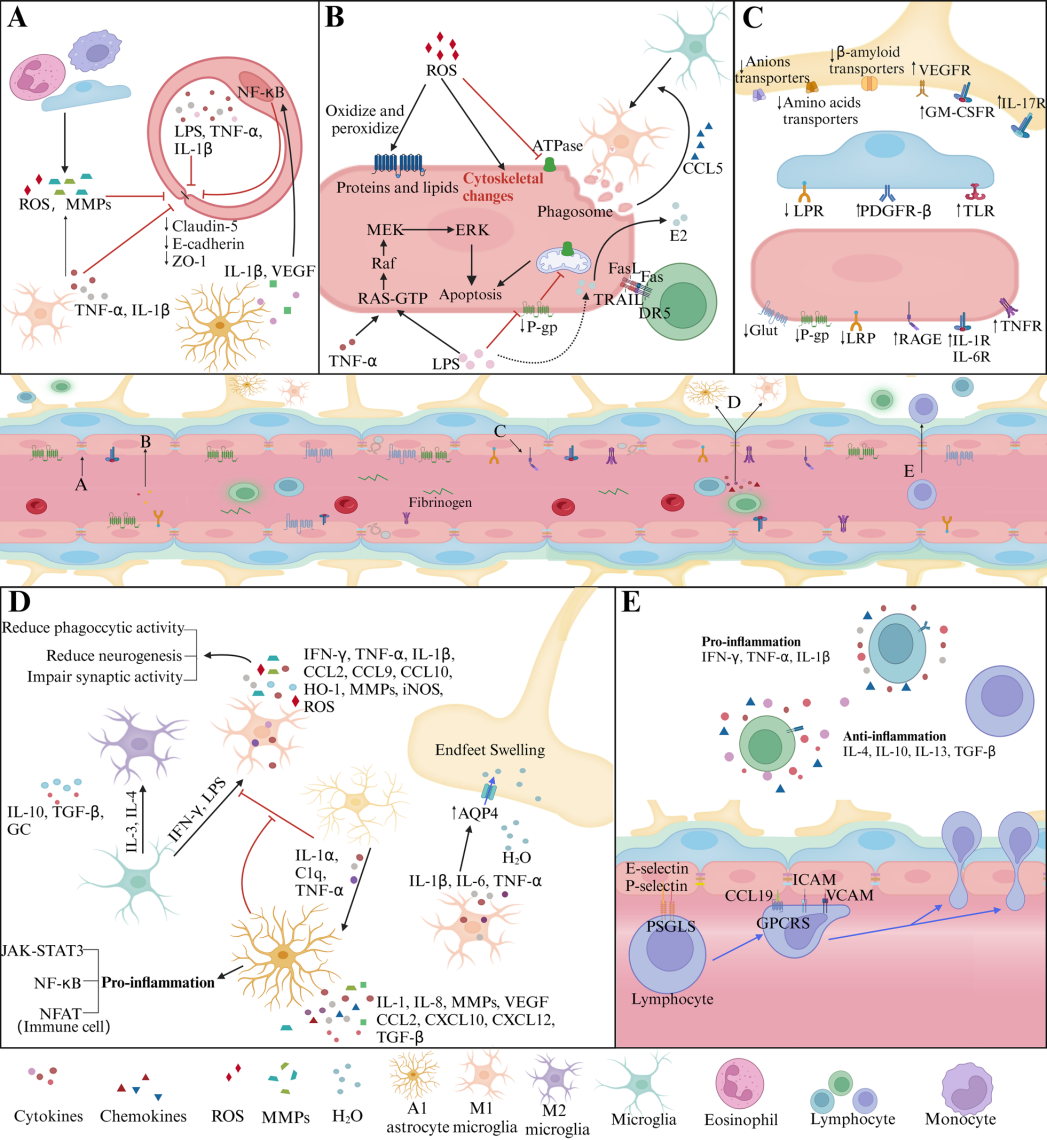
Vascular inflammation. **A** Changes in TJs. Immune cells and molecules like LPS, TNF-α, IL-1β, ROS, and MMPs break TJs, while VEGF from astrocytes weakens them via PLCγ/PKCα/eNOS or PI3K/Akt/eNOS pathways when interacting with VEGFR on ECs. **B** Inflammation affects ECs. ROS disrupts the cytoskeleton, impairs Ca^2+^ influx, and damages membrane proteins, causing EC dysfunction. LPS harms ECs by inhibiting P-gp, promoting prostaglandin E2 synthesis, and triggering EC apoptosis through the MAPK pathway alongside TNF. Microglia recruited by CCL5-CCR5 signaling phagocytose ECs under chronic inflammation, worsening BBB breakdown. Activated T cells target ECs for apoptosis via the Fas pathway. **C** Modifications of transport pathways and receptors. Peripheral inflammation significantly impacts various transport pathways. Efflux transporters (e.g., P-gp) decrease, while influx transporters for insulin, monoamines, and lysosomal enzymes increase. Endothelium displays heightened IL-1, IL-6, RAGE, and TNF-α receptors, and reduced LRP, P-gp, and glutamate transporters. Pericytes downregulate LRP but upregulate PDGFβ and TLR. **D** Activation of Astrocyte and microglia. Astrocytes and microglia react to inflammation differently. Microglia polarize into pro-inflammatory M1 or anti-inflammatory M2 types, affecting synaptic function and neurogenesis. Reactive astrocytes triggered by M1 microglia cytokines lose their regulatory control over microglia, while M2 microglia-secreted cytokines dampen inflammation. Inflammatory cytokines released by microglia induce AQP4 upregulation, causing astrocytic end-feet swelling. **E** Infiltration of immune cells. Immune cell infiltration follows a multistep process involving capture, rolling, adhesion, crawling, and migration. Once infiltrated, lymphocytes adopt pro- or anti-inflammatory roles based on secreted cytokines

#### Astrocyte-mediated regulation of CBF

Perivascular astrocytes, located near synapses, are stimulated by neuronal activity, while their end-feet envelop blood vessels and communicate with SMCs and pericytes, which regulate CBF. The strategic positioning of perivascular astrocytes enables their function as relay cells in the NVU [54]. These astrocytes respond to neuronal glutamate via metabotropic glutamate receptors (mGluR), leading to an increase in intracellular Ca^2+^. This elevation triggers the synthesis of arachidonic acid (AA) in astrocytes. AA then enters SMCs and pericytes and is converted to 20-hydroxy-eicosatetraenoic acid (20-HETE), causing pericyte depolarization and contraction, ultimately reducing CBF [94]. Additionally, elevated extracellular Ca^2+^ and K^+^ levels, either from neuronal activities or astrocytic end-feet, can activate Ca^2+^-gated K^+^ channels, leading to intracellular K^+^ efflux. A moderate increase in extracellular K^+^ concentration (above 10 mM) results in pericyte depolarization and vasoconstriction [8]. AQP4 on astrocytic end-feet relocates towards the soma, causing astrocyte swelling, capillary compression, and decreased CBF [95–97]. The deletion of AQP4 has been shown to improve CBF distribution in cases of brain edema, highlighting its significance in CBF dynamics [98].

**Fig. 5:**
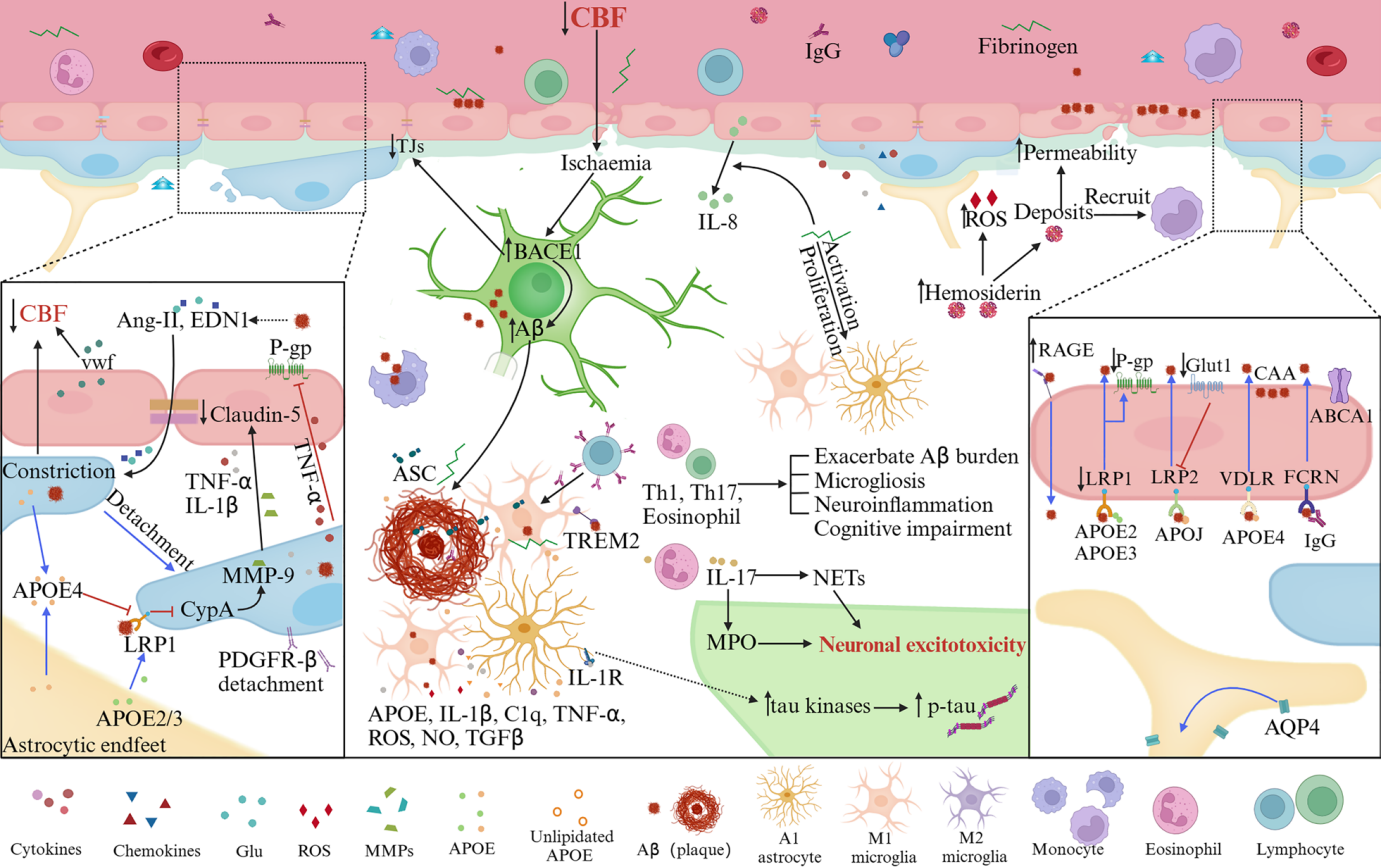
BBB dysfunction in AD. BBB breakdown in AD is characterized by TJ disruption, pericyte degeneration, and imbalanced Aβ clearance/production, impacting neuronal activity. ECs express receptors (LRP1, RAGE, FCRN) and transporters (P-gp, ABCA1) facilitating Aβ transcytosis across the BBB. Aβ complexes with APOE or IgG for enhanced recognition and clearance, with Aβ-APOE4-VLDLR interaction slowing endocytosis. Reduced Aβ clearance due to downregulated LRP1/P-gp and upregulated RAGE coincides with lower Glut1 expression, suggesting EC clearance impairment. Loss of AQP4 in perivascular astrocytes is associated with Aβ accumulation. Astrocytes, neutrophils, and BACE1 regulate Aβ secretion, while APOE4, ASC, fibrinogen, and Tregs contribute to Aβ plaque formation. Microglia cluster around plaques forming a protective barrier, influenced by fibrinogen, which activates EC IL-8. Infiltrated fibrinogen/IgG trigger microglia/astrocyte proliferation and release of various factors, including APOE, C1q, TNF-α, ROS, NO, and TGFβ, with IL-1β potentially phosphorylating tau. Neutrophils infiltrating the brain cause neurotoxicity via IL-17, NETs, and MPO. Aβ-specific CD^4+^ T cells exacerbate Aβ accumulation, microgliosis, inflammation, and cognition decline. Hemosiderin deposits from microhemorrhages boost ROS and vascular permeability, attracting inflammatory monocytes to amyloid deposits, and contributing to CAA. Pericyte-derived TNF-α/IL-1β reduces claudin-5, amplifies infiltration of vasoconstrictors Ang-2/EDN1, and constricts pericytes. Infiltration of Ang-2/EDN1, EC-derived Willebrand Factor (VWF), and pericyte loss reduce CBF. APOE4, mainly from astrocytes/pericytes, weakens APOE2/3 binding to LRP1 and activates CypA–MMP9 pathway, exacerbating BBB instability

#### Pericytes-mediated regulation of CBF

Brain pericytes express contractile and cytoskeletal proteins that can modify CBF by adjusting their contractile tone and diameter [39, 99]. The degree of pericyte loss or degeneration directly correlates with the magnitude of functional changes in the vasculature, underscoring their critical role in preserving cerebral homeostasis. Selective optical ablation of individual capillary pericytes leads to sustained local capillary dilation and a twofold increase in blood cell flux until pericyte contact is regained [5, 4]. Pericyte degeneration reduces both global and individual capillary CBF responses to neuronal and astrocytic stimuli, resulting in abnormal blood perfusion [4, 100]. Pericyte ablation thoroughly leads to acute BBB breakdown and severe loss of CBF [101].

**Fig. 6:**
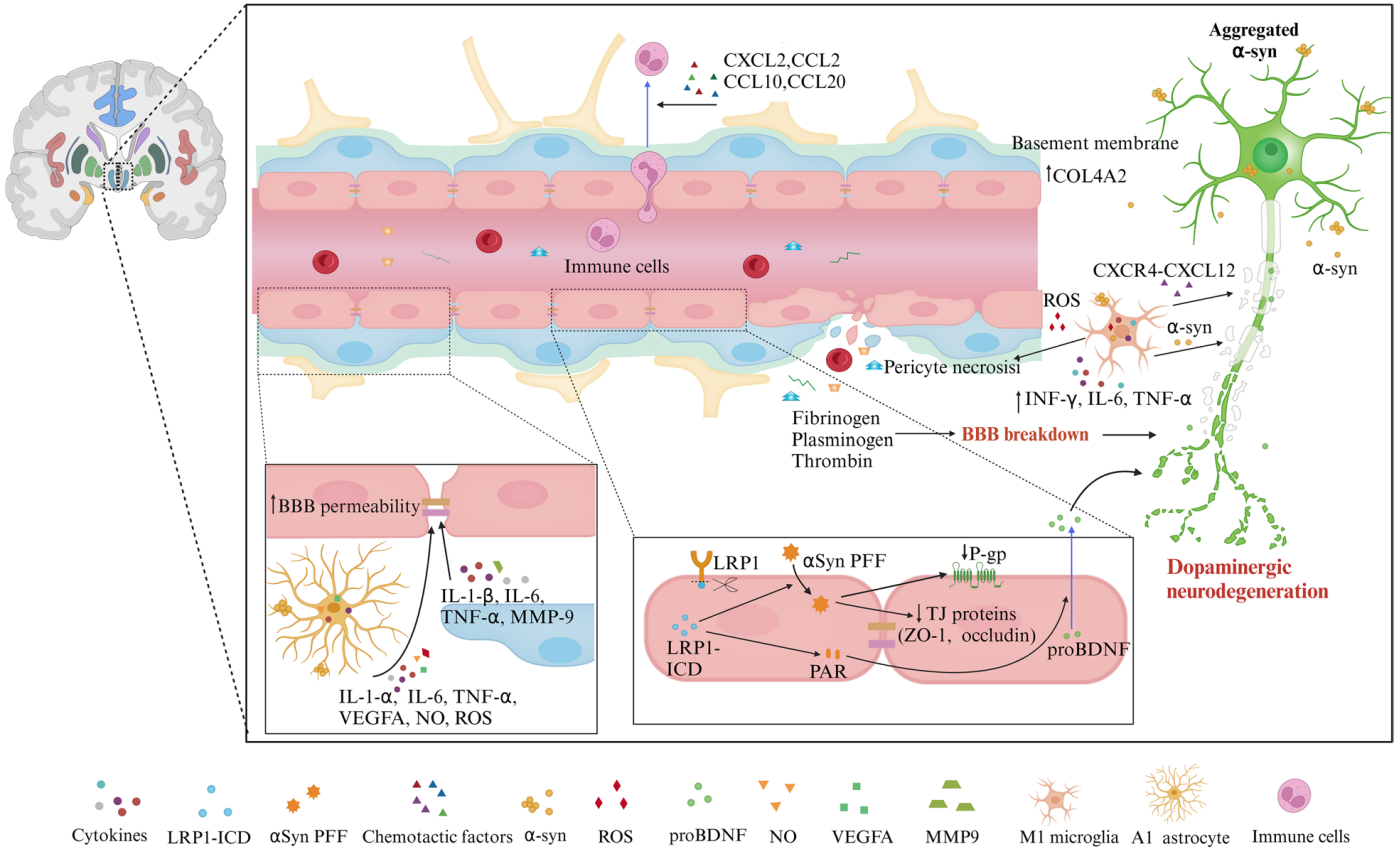
BBB dysfunction in PD. BBB breakdown and dysfunction exist in the basal ganglia of PD patients. In ECs, excessive α-Syn PFFs induce the upregulation of LRP1-ICD, which, in turn, exacerbates αSyn PFF accumulation, and reduces the expression of endothelial TJs such as ZO-1 and occludin, and P-gp. Additionally, endothelial LRP1-ICD increases the expression of protease-activated receptor (PAR), leading to the release of proBDNF, which affects dopaminergic neurons and contributes to early neuronal apoptosis. Enhanced expression of the COL4A2 gene, encoding a subunit of type IV collagen, may alter the morphology and function of the basement membrane. Activated astrocytes secrete VEGFA and NO, causing downregulation of TJs. αSyn PFFs activate pericytes, microglia, and astrocytes, promoting the release of cytokines (IL-1-α, IL-1-β, IL-6, TNF-α, IFN-γ), MMP-9, and ROS, triggering inflammatory reactions and cellular degeneration in ECs, thereby increasing BBB permeability. Upregulated pro-inflammatory chemokines (CCL2, CCL10, CCL20, CXCL2) released by astrocytes attract immune cells from the peripheral circulation into the CNS, exacerbating the inflammatory response. Microglia activated by αSyn PFFs exhibit elevated expression of chemokine receptor CXCR4 and its ligand CXCL12, promoting dopaminergic neuron apoptosis. Furthermore, activated microglia release ROS, leading to pericyte necrosis. The disruption of the BBB allows the entry of plasma fibrinogen, RBCs, and other substances into the brain parenchyma, aggravating pathological processes

The brain capillary pericytes respond to neuronal and astrocytic signals by regulating intracellular Ca^2+^ and K^+^, leading to either hyperpolarization or depolarization, thereby influencing CBF. Neuronal transmitters such as ACh and adenosine bind to their respective receptors on pericytes—MRs for ACh and adenosine A_2A_ receptors for adenosine—causing pericyte hyperpolarization and increased CBF. Activation of large-conductance (BK_Ca_) and small-conductance (K_Ca_) K^+^ channels, as well as ATP-sensitive K^+^ (K_ATP_) channels, induces K^+^ efflux, reducing Ca^2+^ entry through voltage-gated calcium channels (VGCCs), leading to pericyte hyperpolarization and increased CBF [93]. Increases in extracellular K^+^ mediated by neuronal activities, along with vasoconstrictors such as endothelin 1 (ET1), PDGFB, and blood-derived insulin-like growth factor 1 (IGF1), trigger Ca^2+^ entry into pericytes through VGCCs, causing depolarization and contraction [7, 93]. Depolarization of pericytes can also result from neuronal transmitters like NA, ATP, and Ang2 binding to their corresponding receptors on pericytes, as well as from astrocytic AA entering pericytes [102, 103]. Excessive Ca^2+^ entry into the cytosol leads to pericyte calcium overload and contraction, causing microvascular constriction. The resulting death of pericytes leaves the microvasculature continuously constricted, leading to microcirculatory failure and long-term cerebral hypoperfusion due to the contractile apparatus remaining in rigor upon pericyte death, thus reducing capillary diameter [94, 104].

**Fig. 7:**
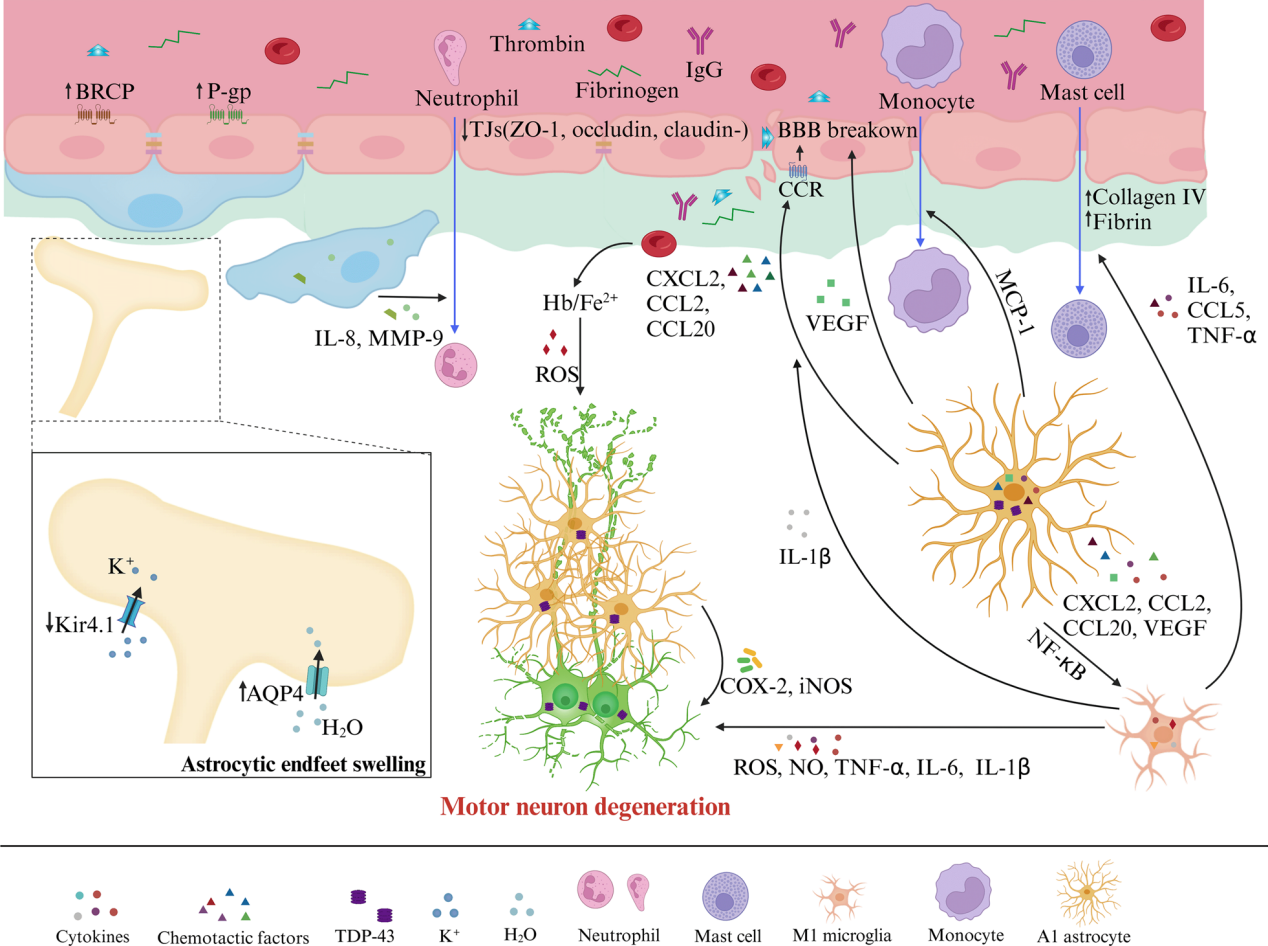
BBB dysfunction in ALS. Hallmark features of ALS include vascular dysfunction and motor neuron degeneration. The BBB breakdown in ALS is characterized by the loss of TJs including ZO-1, claudin-5, and occludin, along with degeneration of pericytes and ECs. This degeneration also involves swelling and detachment of astrocytic end-feet from vessels, elevated levels of BM components such as collagen IV and fibrin deposits, leading to the infiltration of blood-borne cells (red blood cells, neutrophils, monocytes, and mast cells), and plasma-derived proteins (fibrinogen, IgG, and thrombin) into the CNS. In ALS patients, augmented expression and activity of P-gp and BCRP on ECs have been observed. Decreased expression of the potassium channel Kir4.1 and an increased level of AQP4 in astrocytic end-feet are implicated in the swelling of these structures and detachment from the endothelium. Upregulation of TDP-43 in astrocytes promotes microgliosis through the NF-κB pathway. Activated microglia secrete IL-1β, which in turn stimulates astrocytes to release VEGF and pro-inflammatory chemokines including CXCL2, CCL2, and CCL20, resulting in BBB breakdown. Activated microglia contribute to oxidative stress in neurons through the release of ROS, NO, and pro-inflammatory cytokines (TNF-α, IL-6, and IL-1β), leading to motor neuron degeneration. COX-2 and iNOS released by astrocytes surrounding motor neurons, as well as free iron (Fe^2+^) and subsequent ROS production induced by extravasated RBCs, also contribute to this degeneration. Inflammatory reactive pericytes promote neutrophil transmigration via the release of IL-8 and MMP-9. Astrocytes secrete MCP-1, mediating monocyte migration into the CNS. Additionally, activated microglia release IL-6, CCL5, and TNF-α, which lead to the activation and infiltration of mast cells into the CNS

#### Endothelium-mediated regulation of CBF

ECs receive signals from the blood and perivascular cells, including neurons, astrocytes, and pericytes, and play a critical role in regulating CBF. When ACh released from neurons or blood activates endothelial MRs, it triggers an increase in NO production in ECs, leading to cell hyperpolarization and relaxation. ECs contain two types of K^+^ channels: Ca^2+^-activated K^+^ channels (K_Ca_) and inward-rectifier K^+^ channels from the Kir2 family (K_IR_). K_Ca_ channels respond to signals from activated MRs, facilitating K^+^ influx. On the other hand, K_IR_ channels, activated by elevated external K^+^ during neural activity, primarily mediate K^+^ influx without causing cellular hyperpolarization [105]. Instead, they initiate a rapidly propagating retrograde hyperpolarization, transmitting electrical signals to upstream small arteries. This process results in the dilation of these arteries, consequently increasing blood supply to the capillary bed [26, 106].

### Vascular inflammation

The vasculature serves as a vital conduit for nourishment, it inevitably involves the transportation of not only essential nutrients but also potentially harmful substances. Concurrently, the vascular system also grapples with managing the byproducts of oxidative stress generated from both the peripheral circulation and metabolic processes within the brain tissue. An instant vascular inflammatory response, though initially protective against such threats, can become detrimental if excessive or chronic. For example, preconditioning with low doses of lipopolysaccharides (LPS), a component of bacteria cell walls known to trigger an immune response, has demonstrated neuroprotective effects including enhancing cellular resilience, supporting neuronal health, and promoting a monocyte phenotype that aids in tissue repair rather than causing damage [27, 107, 108]. The BBB can resist leakage during systemic inflammation, especially at low levels, which is partly attributed to its robust efflux system, which plays a crucial role in maintaining the brain’s protected environment [109, 110]. These observations suggest that mild inflammatory stimuli can activate endogenous defense mechanisms that strengthen the BBB and the overall CNS resilience. However, when inflammation escalates beyond a certain threshold, vascular inflammation can compromise the structure and function of the BBB, playing a pathological role in multiple CNS diseases [110]. The following section will examine how vascular inflammation induced by BBB disruption manifests in various CNS disorders (Fig. 4).

Inflammation adversely affects the BBB and neural transmission by escalating “inflammatory” signals such as cytokines and chemokines, and by mobilizing “inflammatory” cells including macrophages, neutrophils, and lymphocytes. These elements disrupt homeostasis and exert inflammatory effects on ECs. As the primary barrier, ECs are pivotal targets for peripheral inflammatory factors. These factors, including lipopolysaccharide (LPS), tumor necrosis factor-alpha (TNF-α), and interleukin-1 beta (IL-1β), may impede P-gp activity, activate the mitogen-activated protein kinase (MAPK) signaling pathway, and increase nuclear factor kappa-light-chain-enhancer of activated B cells (NF-κB) expression in ECs. This cascade induces endoplasmic reticulum stress and mitochondrial damage in ECs, leading to apoptosis [111, 112]. Additionally, ECs can respond to IL-1β in the bloodstream by synthesizing and releasing prostaglandin E2, which influences prostaglandin receptors on neurons and glial cells, potentially leading to CNS diseases [113]. Enhanced BBB permeability can be seen as a result of peripheral inflammation, mediated by alterations in the expression of TJs and transporters. “Inflammatory” signals, including IL-1β, IL-6, IL-9, IL-17, interferon-gamma (IFN-γ), TNF-α, and chemokine ligand 2 (CCL2), contribute to diminished TJs expression or misallocation [114–119]. Additionally, there are indirect factors influencing TJs, such as matrix metalloproteinases (MMPs), NO, ROS, Rho-associated protein kinase (ROCK), and NF-κB signaling pathways [120–124]. In addition to ECs, pericytes and astrocytes are also activated in response to peripheral inflammation, significantly impacting BBB permeability. These “inflammatory” signals further affect BBB transport systems, which regulate molecular exchanges between the blood and the brain. Specifically, certain efflux transporters like P-gp on astrocytic end-feet, as well as transporters for anions, amino acids, and β-amyloid, are downregulated. Conversely, influx transporters for insulin, monoamines, and lysosomal enzymes undergo upregulation [125]. Additionally, inflammatory signals lead to increased expression of adhesive molecules on ECs, including vascular cell adhesion molecule 1 (VCAM-1), intercellular adhesion molecule 1 (ICAM-1), and E-selectin. This elevation facilitates the entry of peripheral immune cells into the CNS, a process observed in aging and chronic inflammation [126–129]. Another adverse effect involves the leakage of toxic blood substances such as albumin, fibrinogen, thrombin, and immunoglobulin G (IgG) into the brain. This extravasation alters the neural environment, potentially triggering CNS diseases. For instance, blood-borne fibrin is known to activate neurotoxic innate immune responses in multiple sclerosis mice and to stimulate neurotoxic microglial programs in AD mice [130].

Leaky vascular signals and immune cells serve as potential activators of the innate immune response in CNS diseases. Innate immune cells, including microglia, effectively integrate and rapidly respond to environmental cues. Microglia possess the capability to detect microorganisms, leukocytes, and their metabolites, such as LPS, granulocyte–macrophage colony-stimulating factor (GM-CSF), and can swiftly initiate an immune response by secreting IL-1β, IFN-γ, and TNF-α. These cells are drawn towards blood vessels by the chemokine CCL5, released by ECs, which also plays a role in the production of claudin-5. Activation of glial cells leads to the release of inflammatory cytokines, including IL-1β, IL-6, and TNF-α. This process induces the upregulation of AQP4 and causes swelling in the astrocytic end-feet [131]. Furthermore, IL-1α, TNF-α, and complement component subunit 1q (C1q) encourage the transformation of astrocytes into reactive astrocytes (A1), subsequently impairing the astrocytic capacity to regulate microglial activity [132]. Following this, activated microglial cells adhere to ECs, a process mediated by macrophage-1 antigen (Mac-1). The release of IL-1β and TNF-α can lead to a reduction in the expression of TJs and facilitate the engulfment of ECs by microglial cells [133, 134]. In the later stages of stroke, microglial cells contribute to the remodeling of the ECM by secreting MMPs such as MMP-3, MMP-9, and MMP-19 [135]. The breakdown of the BBB allows infiltrating leukocytes, including monocytes, B cells, T cells, and neutrophils, to be recruited to the CNS [136]. These leukocytes secrete various cytokines that can either exacerbate or mitigate inflammatory responses.

## BBB dysfunction in neurodegenerative diseases

### AD

In the early stages of AD, there is clear evidence of both disruption and dysfunction within the BBB [26]. Disruption of the BBB including compromised BBB integrity and subsequent leakage of plasma constituents into the brain parenchyma, and impaired BBB dysfunction consisting of compromised transport system, reduced CBF, and heightened vascular inflammatory response at the BBB level, which are closely interlinked with the accumulation of Aβ and the presence of neurofibrillary tangles [137]. Emerging evidence suggests that declines in CBF and vascular dysfunction may precede other established AD biomarkers, including Aβ, amyloid, and tau proteins (Fig. 5) [26, 138].

#### Impaired BBB integrity

Postmortem studies of AD patients and animal models have revealed cellular infiltration, including red blood cells [139], macrophages [140], and neutrophils [141], as well as capillary leakage of blood-derived components such as fibrin(ogen), IgG, albumin, thrombin, and hemosiderin [142–145]. These findings indicate compromised integrity of the BBB. Before obvious clinical symptoms appear in AD patients, increased BBB permeability occurs in many brain regions, including the hippocampus, cortex, deep gray matter, and white matter by Dynamic contrast-enhanced magnetic resonance imaging (DCE-MRI) [146–149]. The deterioration of BBB integrity is further correlated with pericyte degeneration and apoptosis, as evidenced by the presence of CSF PDGFRβ, a pericyte marker, and the detachment of PDGFR-β from pericytes [146]. As a significant genetic risk factor for sporadic AD, the Apolipoprotein E (APOE4) allele demonstrates a reduced affinity for LRP1 in pericytes. This reduction activates the Cyclophilin A (CypA)-MMP-9 pathway, which is responsible for the breakdown of the BBB, degeneration of pericytes, and loss of CBF [143, 150–152]. Furthermore, APOE4 is associated with a decrease in the basal membrane area and an increase in thrombin concentrations in the vessel walls [144, 153].

#### Impaired transport system

Impaired glucose metabolism is a prominent characteristic of AD [154, 155]. A reduction in Glut in the ECs of AD patients highlights the compromised function of the BBB [156–158]. Patients with mild cognitive impairment (MCI) exhibit decreased glucose absorption [159]. Fluorodeoxyglucose-PET imaging shows characteristic hypo-metabolism in the posterior cingulate cortex and precuneus in the early stages of Alzheimer's disease, with temporoparietal hypo-metabolism manifesting before structural changes [160]. As AD progresses, this diminished uptake of glucose becomes increasingly apparent in regions such as the hippocampus, parietal cortex, cingulate cortex, and temporal cortex [161, 162]. Glut1deficiency suppresses the expression of low-density LRP1 and reduces CBF, suggesting a connection between CBF, Aβ clearance, and glucose metabolism disturbances [163]. While phosphorylated tau (p-tau) does not directly affect Glut1, it triggers widespread neuronal death, leading to a decline in D-glucose transport in p25 mice [164]. Tau-induced activation of glial cells increases the expression of EC adhesion molecules, facilitating leukocyte migration across the BBB [165]. Although a specific transporter for p-tau has yet to be identified, the involvement of tau pathology in BBB dysfunction necessitates further investigation.

Aβ is primarily cleared through transport-mediated processes, notably via the low-density LRP [166]. Apolipoprotein J (APOJ) binds to Aβ, facilitating its transport across the BBB through the LRP2 receptor [167]. P-gp not only exports Aβ from BBB endothelium but also plays a role in clearing it from neurons [168]. Accumulated Aβ peptides can disrupt these clearance processes by reducing P-gp expression and activity [169–171]. Additionally, Aβ can be eliminated through interstitial fluid (ISF) flow in perivascular spaces [172]. However, Aβ plaques in these spaces can lead to reduced expression of AQP4 at the astrocytic end-feet, adversely affecting Aβ efflux with the ISF and exacerbating intraparenchymal Aβ deposition [58, 173, 174]. Other factors that can hinder Aβ clearance include extracellular proprotein convertase subtilisin/kexin type 9 (PCSK9) impacting LRP1 on ECs and altered expression of β-site amyloid precursor protein cleaving enzyme 1 (BACE1) [175, 176].

#### CBF reductions

Individuals and animals with AD exhibit reductions in CBF and impaired cerebrovascular reactivity, particularly in the hippocampus, thalamus, frontal, and occipital cortices, as assessed by arterial spin labeling magnetic resonance imaging [157, 177, 178]. Decreased CBF precedes the detectable changes of classical Aβ and tau biomarkers, as well as brain atrophy, suggesting that reduced CBF could be a valuable biomarker in preclinical AD [138, 179]. Pericyte degeneration, which can diminish capillary CBF responses to neuronal stimulation, has been reported. Furthermore, pericyte degeneration and apoptosis, as evidenced by the detachment of PDGFR-β from pericytes, are induced by accumulated Aβ and impaired Aβ clearance, leading to reduced CBF [45, 74, 146, 180]. In human brain cortex slices from AD patients, excessive Aβ triggers the production of ROS, consequently elevating intracellular ET1 levels. This elevation activates the endothelin-A receptor on pericytes, causing pericyte contraction and capillary constriction, which ultimately leads to decreased CBF [15]. Aβ also stimulates the activation of NADPH oxidases (NOXs), integrin Iib-3, and ROS, promoting platelet adhesion, increasing levels of the endogenous inhibitors plasminogen activator inhibitor-1 and endothelin-1 (ET-1), and resulting in thrombosis, capillary constriction, and impaired blood flow [99, 181, 182]. There is evidence of a negative correlation between tau protein expression and CBF [183]. Blood vessels in AD exhibit structural irregularities, such as twisting and varying diameters, and may develop cerebral amyloid angiopathy (CAA), significantly contributing to BBB disruption, diminished CBF, and cognitive deficits [19, 94, 184–186]. Furthermore, chronic overproduction of TGFβ1 from astrocytes and microglia leads to decreased pericyte coverage, facilitating vascular remodeling and suboptimal brain perfusion [187–189].

Cerebrovascular reactivity is indicative of the cerebrovascular system's capacity for contraction and dilatation in response to stimuli, such as variations in cerebral perfusion pressure and arterial CO_2_ partial pressure levels. This reactivity reflects the overall functionality of the cerebrovascular system [190]. The reduction in CBF and the diminished vasodilatory response of cerebral vessels to CO_2_, due to the accumulation of Aβ, suggest compromised cerebrovascular reactivity examined by transcranial Doppler and the blood oxygenation level-dependent (BOLD) functional MRI (fMRI) [191–193]. MRI research has disclosed a diminished CBF response to visual stimulation and during memory encoding processes specifically in the hippocampal region of AD patients [194, 195]. Additionally, pathogenic soluble tau proteins impair endothelium-dependent CBF responses, leading to microvascular deficits. These deficits are mediated by EC senescence and inhibited activation of endothelial nitric oxide synthase (eNOS), which is exacerbated by the internalization of soluble tau [196].

#### Vascular inflammatory response

The disruption of the BBB can lead to inflammation that exhibits a dual nature. In acute inflammation phases, this disruption may offer protection in microglial cells, but it becomes detrimental during chronic responses [84]. In the early stages of AD, heightened immune responses facilitate the clearance of Aβ [197, 198]. However, prolonged inflammation impairs the microglial cells' ability to bind and phagocytose Aβ, diminishes the activity of Aβ-degrading enzymes, and weakens their capacity to disintegrate Aβ plaques [199, 200]. Peripheral macrophages, attracted to Aβ plaque deposition sites by CCL2, assist in Aβ clearance, but CCL2 simultaneously exacerbates NVU injury [201–203]. M1 microglia release cytokines such as CCL2, CXCL10, TNF-α, IL-1β, and IL-6, which lead to a decreased expression of TJs in blood vessels [203–206]. While TNF-α does not affect P-gp protein levels, it inhibits its efflux transport activity [207, 208].

Extravasated plasma components act as potent stimulators of inflammatory chemokines, triggering the activation of microglia and astrocytes. Conversely, they damage the BBB and exacerbate the pathology [209–212]. Fibrinogen infiltration, co-localizing with Aβ, promotes IL-8 release by ECs. This alters EC properties through the production of a broad spectrum of proteases and coagulation factors, which disrupt NVU cells and contribute to BBB dysfunction, leading to prolonged inflammation [211, 213–215]. The complexities of how BBB dysfunction is linked with extravasated plasma components, including fibrinogen, IgG, albumin, thrombin, and hemosiderin, remain to be fully elucidated [216].

### PD

PD is primarily attributed to the degeneration and death of dopaminergic neurons in the substantia nigra pars compacta, accompanied by the accumulation of Lewy bodies and Lewy neurites, predominantly comprising α-synuclein, in degenerating neurons [217]. Vascular risk factors have been identified as accelerating the onset and severity of motor and cognitive impairments in PD [19]. Clinical investigations using DCE-MRI reveal a stark contrast in BBB performance between healthy individuals and patients diagnosed with PD. PD patients exhibit pronounced BBB dysfunction, evidenced by heightened leakage, notably in the substantia nigra and striatum pivotal to the disease's pathology [218, 219]. This BBB dysfunction in PD is linked to the degeneration of dopaminergic neurons, which release alpha-synuclein (αSyn) into the brain parenchyma, exacerbating BBB dysfunction (Fig. 6) [220].

In patients with PD, ECs in the basal ganglia undergo degeneration, accompanied by a reduction in the expression of TJs including occludin, and ZO-1 [221]. Decreased expression of P-gp, which participates in the clearance of toxic substances, has been observed in PD patients’ ECs, with dysfunction linked to membrane LRP1 [222, 223]. In PD mouse models, the intracellular domain of LRP1 (LRP1-ICD), released through γ-secretase and matrix metalloproteinase cleavage, accumulates within cells. This leads to the entry and aggregation of alpha-synuclein-preformed fibrils (αSyn PFFs), resulting in the loss of occludin and decreased P-gp expression [224]. Under the influence of αSyn PFFs, LRP1-ICD interacts with poly (adenosine 5'-diphosphate-ribose) polymerase 1 (PARP1), upregulating PAR expression and promoting the secretion of pro-brain-derived neurotrophic factor (proBDNF) by ECs into dopaminergic neurons. This interaction accelerates the damage caused by αSyn PFFs to both ECs and neurons [225, 226]. As an important mechanism of PD neurogenesis, LRP1-ICD/PARP1 provides a vascular perspective for PD treatment. Pericytes activated by αSyn in PD patients express high levels of IL-1β, IL-6, TNF-α, and MMP-9, exacerbating BBB disruption [227–229]. Alpha-synuclein induces activated astrocytes to release VEGF-A and NO, leading to vascular damage and the downregulation of TJs [228, 229]. Elevated expression levels of collagen IV in the BM have been identified in the post-mortem brains of PD patients. Early-stage PD patients and 6-hydroxydopamine (6-OHDA)-treated rodent models exhibit aberrant angiogenesis [230, 231]. This correlates with the significant disruption of the capillary network observed in the substantia nigra [232]. The BBB disruption, indicated by microhemorrhages [26], allows the entry of fibrinogen, plasma proteins, RBC, and other toxic substances into the brain parenchyma, further damaging neurons [233]. Furthermore, MRI studies have revealed microbleeds in both gray and white matter regions of PD patients [234]. Clinical research has demonstrated that a reduction in CBF occurs during the initial phases of the disease, suggesting a connection to αSyn pathology and the aberrant activation of pericytes [27, 235, 236].

The aggregation of αSyn activates microglia and astrocytes, promoting the release of inflammatory mediators and contributing to the degeneration of ECs and neurons. Direct injection of αSyn into the substantia nigra of model rats induces microglial activation, upregulates inflammatory mediators, and increases the expression of chemokine receptor CXCR4 and its ligand CXCL12. This CXCR4-CXCL12 signaling pathway activates Caspase-3, leading to apoptotic neuronal death [237]. Additionally, activated microglia promote pericyte apoptosis through the production of ROS [238]. In the astrocytes of patients with PD, αSyn predominantly accumulates in astrocytic end-feet, particularly within lysosomes. These activated astrocytes secrete numerous inflammatory factors, including TNF-α, IL-1β, and IL-6 [188]. In response to inflammatory mediators, ECs produce pro-inflammatory cytokines and chemokines such as IL-1β, IL-6, CCL2, and CXCL1, and synthesize ICAM-1 and VCAM-1, facilitating the infiltration of immune cells [239].

### ALS

ALS is characterized by the degeneration of motor neurons in the spinal cord, brainstem, and brain, progressively weakening voluntary skeletal muscles and ultimately leading to paralysis [240]. Disrupted BBB and the blood-cerebrospinal fluid barrier are evident in both sporadic and familial ALS, occurring before the onset of motor neuron degeneration (Fig. 7). Research involving ALS patients and model mice has revealed EC degeneration, characterized by cellular damage, swelling, and cytoplasmic vacuolization, along with diminished levels of TJs such as ZO-1, claudin-V, and occluding [241]. ALS patients exhibit increased expression of BM components like collagen IV, whereas model mice show a reduction in collagen IV [241, 242]. The BBB disruption is associated with leakage of blood-derived proteins, including IgG, thrombin, hemoglobin, hemosiderin, and fibrinogen into the parenchyma, and increased IgG, albumin, and complement protein C3a levels in the CSF of ALS patients [46, 240]. MRI examinations have uncovered microbleeds in the deep layers of the motor cortex in ALS patients, while the precise mechanisms driving this BBB impairment in ALS remain elusive [243]. Iron (Fe^2+^) derived from hemoglobin metabolism and immune responses, implicated in early ALS pathogenesis via oxidative stress, contributes to motor neuron damage, exacerbating BBB permeability changes [244, 245].

Significant alterations in the BBB transport systems have been observed in ALS. Increased expression and activity of P-gp in ECs and BCRP in reactive astrocytes are evident in the motor cortex of ALS patients, potentially contributing to multidrug resistance [246]. Notably, a reduction in the potassium channel Kir4.1 and an increase in AQP4 in the astrocyte end-feet of ALS model rats indicate challenges in maintaining water and potassium balance, further compromising BBB stability [247].

Astrocytes surrounding degenerating motor neurons show increased expression of inflammatory markers, notably cyclooxygenase-2 (COX-2) and inducible nitric oxide synthase (iNOS) [248]. TDP-43, accumulating in glial and neuronal cytoplasm, is associated with immune and neuroinflammatory processes [249]. In cultured astrocytes, TDP-43 elevates IL-1β, IL-6, and TNF-α levels, mirroring ALS patient blood profiles and stimulating microglial proliferation via NF-κB activation [250, 251]. Overexpression of TDP-43 in ALS results in the infiltration of immune cells such as CD^3+^ T cells, CD^4+^ T cells, and monocytes, along with IgG leakage, activation of EC and pericyte [240, 252, 253]. Activated microglia release IL-1β, which stimulates astrocytes to secrete VEGF and proinflammatory chemokines like CXCL2, CCL2, and CCL20, further exacerbating BBB dysfunction [240]. Additionally, activated microglia contribute to oxidative stress through the release of ROS, NO, and pro-inflammatory cytokines such as TNF-α, IL-6, and IL-1β, inducing motor neuron death [254].

Peripheral immune cells, initially activated within the peripheral immune system, subsequently transition to the CNS, facilitating the proinflammatory milieu together with the CNS resident immune cells [255]. Beyond the astrocytic activation by TDP-43, pericytes, astrocytes, and microglia regulate the infiltration of peripheral immune cells. Inflammatory reactive pericytes promote neutrophil transmigration through the release of IL-8 and MMP-9 [256]. Astrocytes secrete monocyte chemoattractant protein-1 (MCP-1), mediating monocyte migration and amplifying neuroinflammation [257]. Activated microglia release IL-6, CCL5, and TNF-α contributing to the activation and infiltration of mast cells [258]. Interactions between resident immune cells in the CNS and peripheral immune cells through immune molecules render inflammation systemic, ultimately leading to the dysfunction of neuromuscular junctions, damage to motor neuron axons, and motor neuron death [255].

In conclusion, impairment of the BBB in ALS involves reduced TJs, degeneration of pericytes and ECs, infiltration of peripheral immune cells, and accumulation of plasma-derived toxic proteins. These changes often precede and exacerbate ALS symptoms.

### MS

Multiple sclerosis is an autoimmune, chronic inflammatory disease of the CNS, hallmarked by demyelination and inflammation due to immune cell invasion into the brain parenchyma via a disrupted BBB [259]. Characteristic hyperintense lesions within white matter, discernible on T2-weighted MRI scans, signify core pathological markers of MS [260]. Gadolinium-enhanced neuroimaging has illuminated that BBB dysfunction is a foundational event in MS pathogenesis, occurring early in the disease process [261]. Intriguingly, MRI evidence of BBB disruption has been noted in seemingly normal white matter even before the emergence of enhancing lesions, as well as in non-enhancing regions, implying that BBB impairment precedes both clinical symptoms and other MRI-evident changes in the disease course.

Indicative markers of BBB breakdown, including elevated albumin quotient (Qalb), increased MMP-9 activity in CSF and serum, and a higher CSF leukocyte count, have been documented across various stages of MS [19]. Inflammation is a constant feature across all MS stages, with immune cell-mediated inflammation being particularly pronounced during the initial acute phases. The infiltration of leukocytes across the BBB necessitates intricate molecular interactions between these cells and ECs [19]. T and B lymphocytes, along with monocytes, are activated in the peripheral compartment before infiltrating the CNS [19, 259]. Recent research employing radiolabeled MMP PET has revealed that heightened MMP activity is localized around active lesions, which are linked to leukocyte infiltration [262]. The transmigration of immune cells across the BBB involves interactions with ECs, changes in endothelial transcellular and paracellular transport, and morphological alterations of the immune cells. Upon entry into the parenchyma, these cells initiate a secondary immune response, releasing inflammatory mediators such as cytokines, chemokines, proteases, and toxic substances, aggravating myelin and axonal damage [263]. Resident glial cells become activated, aiding in antigen presentation to T helper cells. Macrophages, upon activation by Th1 cells, release various cytokines and inflammatory molecules, including TNF-α, proteases, NO, and oxygen-free radicals, causing myelin damage [264]. Th17 and Th1 cells influence astrocyte functions, downregulating neurotrophic factors and upregulating inflammatory cytokines and chemokines [265]. Furthermore, TH17 cells impede the maturation and survival of oligodendrocytes (OLs), as well as induce their apoptosis [266, 267]. CD^8+^ cytotoxic T lymphocytes, activated by antigens presented by oligodendrocytes and neurons, are implicated in demyelination and axonal/neuronal damage [268]. Lymphocytes activate ECs and affect BBB permeability through cytokines like IL-6, IL-17, IL-22, and IFN-γ [266]. Pericytes secrete inflammatory factors and physically detach from ECs, thereby creating a local inflammatory milieu that facilitates the recruitment of peripheral immune cells across the BBB [269]. In the early stages of experimental autoimmune encephalomyelitis (EAE), astrocytes within demyelinating lesions become activated before leukocyte infiltration. They release various factors including TNF-α, IL-1β, IL-6, glutamate, and NO, contributing to oligodendrocyte injury and axonal degeneration. IL-17-mediated signaling in astrocytes promotes the secretion of MMP-3 and MMP-9, further compromising BBB integrity. Activated microglia inflict damage on oligodendrocytes through the release of pro-inflammatory cytokines such as IL-1, IL-6, TNF-α, and interferons, alongside phagocytic activity, and antigen presentation to CD^4+^ T cells via MHC class II [270]. Furthermore, neurons are directly impaired by reactive oxygen and nitrogen species (ROS/NRs) and inflammatory cytokines [270].

MRI studies have revealed global cerebral hypoperfusion at all stages of MS, suggesting a link to hypoxic conditions [271]. Imaging studies using SPECT, PET, and ASL have shown reduced CBF in MS patients across all major subtypes, without reaching ischemic severity [271, 272]. The mechanisms behind this hypoperfusion may involve astrocyte energy metabolism compromise due to lacking β2-adrenergic receptors, and increased vasoconstriction from elevated ET-1 [273–275]. Excess NO production and associated cellular desensitization, along with vascular inflammation and structural changes, also contribute to the complex vasculature-related pathology of MS [276, 277]. Oligodendrocytes are notably susceptible to hypoxia [278], and the oxidative stress resulting from chronic hypoxia is a critical mechanism in neurodegeneration and axonal loss in multiple sclerosis. Extravasation of plasma albumin, occurring before cellular inflammation and clinical symptoms, contributes to damage in myelin and oligodendrocytes [279]. Albumin triggers inflammatory responses through astrocytic and microglial expression of IL-1β, CXCL3, and NO. Additionally, albumin’s interaction with TGF-β receptors on astrocytes affects calcium concentrations [280] and leads to the downregulation of Kir4.1. Albumin uptake in neurons heightens NMDA receptor excitability, provoking glutamate excitotoxicity that exacerbates myelin and oligodendrocyte injury, culminating in neuronal apoptosis [281, 282]. Glutamate accumulation, caused by release from activated microglia and lymphocytes and impaired clearance by resident cells like astrocytes, further exacerbates neuronal damage [283]. In the CNS, zinc released from nerve endings activates the MAPK pathway, inducing neuronal apoptosis, MMP-9-dependent BBB disruption, and microglial activation, which in turn releases proinflammatory cytokines, ultimately damaging the myelin sheath [284].

## Mechanisms affecting BBB to neurodegeneration

Neurodegenerative diseases such as AD, PD, ALS, and MS, while distinct in clinical presentation, share a convergence on impaired BBB stability, CBF reduction, and vascular inflammation, reflecting parallel paths of progressive neurodegeneration distinct from acute brain injuries.

### Impaired BBB stability

DCE-MRI has exposed increased regional BBB permeability in the cortex and hippocampus in AD, basal ganglia in PD, and white matter in MS. Increased BBB permeability suggests loss of BBB integrity evidenced by EC degeneration, TJs decrease, and pericyte deterioration and detachment from ECs. Enhanced permeability of the BBB and subsequent leakage of blood contents exacerbate BBB disruption, fueling a vicious cycle detrimental to the imbalance of the CNS, ultimately contributing to neuronal degeneration, and driving neurodegenerative disorders pathophysiology.

### CBF reduction

ASL-MRI studies indicated region-specific decreased CBF in neurodegenerative disorders like AD, PD, and MS. Decreased CBF observed in the early stages of AD, non-demented PD patients, ALS cases before cognitive decline, and in normally appearing white matter of MS patients without cognitive impairments, implies that CBF reduction may serve as an early biomarker, preceding neurodegenerative changes. Perfusion abnormalities hinder the clearance of metabolic byproducts, compromising cerebrovascular health, and enhancing vulnerability to disease initiation. Moreover, CBF reduction directly damages the cerebrovascular system, as evidenced by the widespread degeneration, detachment, and loss of pericytes, phenomena that are consistently observed in regions experiencing reduced CBF in various neurodegenerative diseases [94].

### Vascular inflammation

A ubiquitous feature among neurodegenerative diseases is vascular inflammation, triggered by advancing pathology, BBB compromise, and oxygen deficiency. The vasculature is persistently challenged by blood-borne toxins, leukocytes, and metabolic waste from both the circulatory and the brain. When the integrity of the BBB is breached, infiltrating blood components and immune cells activate perivascular glia, igniting an inflammatory cascade that accelerates neuronal damage. The disease-specific mechanisms stimulate atypical glial cell activity, releasing a cascade of inflammatory mediators that target blood vessels, further deteriorating the BBB. In later stages, heightened central nervous system inflammation fuels disease progression and exacerbates BBB dysfunction, with heightened levels of enzymes like MMP-9 exacerbating this breakdown.

The distinctive clinical manifestations and singular pathophysiological mechanisms underpinning AD, PD, ALS, and MS engender unique profiles of BBB dysfunction tailored to each disorder. In AD, the excessive accumulation of Aβ overwhelms clearance mechanisms, leading to an imbalance implicated in BBB impairment. The APOE ε4 allele, a significant genetic risk factor for sporadic AD, contributes to BBB dysfunction and exacerbates AD pathogenesis through the APOE4-CypA-MMP9 pathway. In PD, disruptions to the BBB facilitate the spread of αSyn within the brain, while αSyn aggregates reciprocally worsen BBB damage by instigating immune reactions or compromising BBB integrity, perpetuating a detrimental feedback loop that accelerates PD's pathology. The progression of ALS is driven by a complex interplay among astrocyte abnormalities, chronic neuroinflammation, and TDP-43 aggregation, forming a unique web of interconnected pathologies. Astrocyte dysfunction, beyond disrupting water and potassium homeostasis through altered AQP4 and Kir4.1 expression, amplifies neuroinflammation around degenerating motor neurons, exacerbating their deterioration. MS is marked by persistent, chronic inflammation throughout its progression, characterized by immune cell attacks on the myelin sheath. Leukocytes, T lymphocytes, B lymphocytes, and macrophages infiltrate the CNS via a compromised BBB, targeting myelin and perpetuating the cycle of inflammation and demyelination.

## Therapeutic implications and future directions

Currently, the available evidence demonstrates that region-specific BBB dysfunction including reduced CBF (shortfalls and dysregulations), impaired BBB integrity (increased BBB permeability and transport system), and vascular inflammation (extravasated plasma components and peri-vascular inflammatory responses) contributes to the onset and progression of multiple neurodegenerative disorders. Undoubtedly, treatment for BBB disruption shows great potential for neurodegenerative diseases by regulating the pathological processes [24]. The subsequent sections elucidate methods for restoring the structural and functional BBB.

### Normalizing barrier function

Various pharmacological agents have shown efficacy in protecting against neurodegenerative diseases by enhancing BBB integrity. Activated Protein C (APC), which cleaves protease-activated receptor 1 in brain endothelium and targets Rac1 GTPase, strengthens the BBB via the β-arrestin-2-dependent biased signaling pathway [285]. APC has also demonstrated therapeutic potential in models of AD, ALS, and multiple sclerosis by reducing BBB breakdown, inflammatory responses, and neuronal damage [286–290]. In murine models, cyclosporine, functioning as a CypA inhibitor, diminishes the CypA-NF-κB-MMP-9 pathway associated with BBB degradation and neurodegenerative changes. This results in the restoration of BBB integrity and the reversal of neuronal dysfunction and degeneration [291].

BBB breakdown is often accompanied by the loss of TJs. Several pharmacological agents targeting TJs have demonstrated their efficacy in enhancing BBB functions. 10-O-(N, N-dimethylaminoethyl) ginkgolide B methanesulfonate (XQ-1H) has been shown to normalize the expression of claudin-5, occludin, ZO-1, and β-catenin in ischemic stroke model mice, leading to reduced BBB permeability and protection against oxygen–glucose deprivation/reoxygenation injuries [292]. In intracerebral hemorrhage mouse models, the normalization of tight junction proteins ZO-1 and occludin by fibroblasts has been observed to enhance BBB integrity [293]. Additionally, emerging research on gut-derived microbial metabolites and induced pluripotent stem cells (iPSCs) offers new therapeutic directions. For instance, mouse models lacking gut microbiota exhibit diminished expression of occludin and claudin-5, along with increased BBB permeability [294]. Mesenchymal cell transplantation has also shown promise in vascular regeneration therapy and BBB repair [295, 296]. Importantly, methods for drug delivery involving transient disruption of TJs are emerging as potential treatment strategies.

### Normalizing clearance function

The BBB serves as a crucial clearance site for various brain-originated toxic substances within the CNS, particularly for the removal of Aβ in AD and α-synuclein in PD. Low-density LRP1 on ECs facilitates the trans-endothelial clearance of these neurotoxins [297]. Enhanced Aβ clearance, achieved through the delivery of the LRP1 minigene to the BBB via viral vectors, has been shown to reduce the accumulation of Aβ in the brain parenchyma and alleviate Aβ pathology [19]. Additionally, α-synuclein, found in Lewy bodies of both Lewy body dementia and PD, can also be cleared from the brain via LRP1-mediated transcytosis [298]. Animal studies have revealed that allopregnanolone promotes the clearance of Aβ and cholesterol [299]. Inhibiting RAGE may suppress Aβ accumulation in the brain parenchyma, and small molecule RAGE inhibitors are currently advancing to clinical trials [19, 300]. Thus, enhancing the BBB's ability to eliminate pathogenic factors that accumulate in the CNS holds considerable promise for improving outcomes in neurodegenerative diseases.

### Targeting vascular inflammation

A crucial approach for treating vascular inflammation involves controlling pro-inflammatory cytokines, MMPs, and infiltrating leukocytes [301]. Glucocorticoids, long regarded as the frontline therapy for inflammatory conditions, bolster BBB integrity by enhancing TJs and suppressing MMPs and inflammation [302]. Resolvin D (RvD) has demonstrated promise in reducing inflammation by decreasing leukocyte-EC interactions [303]. Furthermore, astrocytes and microglia are gaining recognition as pivotal therapeutic targets in neuroinflammation. Regulating astrocyte reactivity through metabolic pathways has proven effective. For instance, glucosylceramide synthase inhibitors can mitigate neuroinflammation by interrupting immunometabolic pathways in pathogenic astrocytes [304]. Minocycline acts by inhibiting MMP-9 and the p38 mitogen-activated protein kinase signaling pathways, thereby reducing the production of glutamate, IL-1β, and NO by microglia [305]. Despite these advances, it is imperative to recognize the limitations encountered in clinical trials of anti-inflammatory medications aimed at vascular inflammation. High doses of glucocorticoids have adverse outcomes in stroke interventions, as they impair EC barrier function [306]. Similarly, endeavors in clinical research to mend the BBB through attenuation of brain inflammation or the targeting of cerebral Aβ in AD patients have, to date, proven unsuccessful [307]. These setbacks may stem from the swift degeneration of neurons, supportive glial cells, and cerebral vasculature post-trauma, complicating the management of inflammation in injured brains [308]. Navigating the landscape of vascular inflammation therapy is fraught with obstacles, yet it concurrently harbors untapped molecular pathways brimming with potential.

Recent advancements in research have greatly enhanced our comprehension of BBB dysfunction and furnished us with strategies aiming at restoring the structural and functional BBB in neurodegenerative diseases. Nonetheless, opportunities and challenges remain regarding the BBB in the context of diseases. First, how can we identify the degree of BBB breakdown and its intricate connection to neurodegenerative diseases within a clinical setting? The bulk of clinical evidence about BBB breakdown is derived from postmortem human brain tissue analyses, which inherently limits our ability to assess real-time dysfunction or track disease progression [27, 309]. Moreover, there is an inadequate availability of preclinical models effectively exhibiting BBB dysfunction characteristics akin to those observed in neurodegenerative conditions [12, 310]. This deficiency poses an obstacle to monitoring or discerning such dysfunction as a measurable outcome in vivo studies concerning pathology. Besides, the precise correlation between varied functional alterations in distinct brain regions affected by different neurodegenerative disorders and the corresponding modifications in brain vasculature and CBF within those areas remains elusive and not clearly defined. As for the research of inflammatory response, how significant is the role of the BBB in mediating the effects of systemic inflammation on neurological disease progression needs more evidence to demonstrate. Despite there are quantity of BBB-targeted treatment strategies based on animal models, the situation is disappointing when used in clinical trials for the limited rate of success. The specificity and effectiveness of related treatment strategies should be strengthened in the subsequent research.

## Conclusion

The recognition of BBB dysfunction as a common feature in multiple neurodegenerative disorders has underscored the importance of maintaining healthy blood vessels for optimal neural function. Understanding the specific manifestations and shared mechanisms of BBB breakdown in prevalent neurodegenerative diseases opens new research and therapeutic opportunities. This review provides a comprehensive overview of the interplay between vascular stability, CBF regulation, and vascular inflammation in the context of the BBB, with a particular emphasis on the dysfunction of these mechanisms in the pathophysiology of neurodegenerative diseases such as AD, PD, ALS, and MS. It underscores the critical role these factors play in disease progression and highlights the importance of understanding their complex interactions for the development of novel therapeutic strategies aimed at preserving BBB integrity and mitigating neurodegeneration. Recent therapeutic strategies targeting BBB deterioration and related neurodegenerative conditions focus on enhancing drug delivery to the CNS and restoring BBB integrity. However, current research on the BBB and methods for repairing it are predominantly limited to animal models. Future studies are needed to elucidate the molecular and cellular aspects of human brain vasculature in health and disease. The application of single-cell RNA sequencing and advancements in neuroimaging, such as PET imaging and MRI, are invaluable for understanding the molecular and cellular underpinnings of BBB dysfunction. These approaches also aid in identifying novel vascular biomarkers and are essential in developing effective therapies for various neurological diseases.

## Data Availability

Not applicable.

## Abbreviations

BBB: Blood–brain barrier
CNS: Central nervous system
NVU: Neurovascular unit
ECs: Endothelial cells
CBF: Cerebrovascular blood flow
TJs: Tight junctions
AJs: Adherens junctions
JAMs: Junctional adhesion molecules
ZO: Zonula occludin
VE-cadherin: Vascular endothelial cadherin
PECAM-1: Platelet endothelial cell adhesion molecule-1
CMT: Carrier-mediated transport
RMT: Receptor-mediated transport
Mfsd2a: Major facilitator superfamily domain containing 2a
Glut1: Glucose carrier 1
LRP1: Lipoprotein receptor 1
LRP2: Lipoprotein receptor 2
RAGE: Advanced glycation end products
ABC transporters: ATP-binding cassette transporters
P-gp: P-glycoprotein
BCRP: Breast cancer resistance protein
ICAMs: Intercellular adhesion molecules
Aβ: Amyloid-beta
AQP4: Aquaporin-4
PDGF-BB: Platelet-derived growth factor-BB
PDGFRβ: PDGF receptor-β
VEGF-A: Vascular endothelial growth factor-A
VEGFR2: VEGF receptor 2
TGF-β: Transforming growth factor-β
TGFβR2: TGF-β receptor 2
Ang1: Angiopoietin-1
Ang2: Angiopoietin-2
SMCs: Smooth muscle cells
A2AR: Adenosine A2A receptors
NA: Noradrenaline
α2A: α2-Adrenergic receptors
ACh: Acetylcholine
MRs: Muscarinic ACh receptors
NO: Nitric oxide
mGluR: Metabotropic glutamate receptors
AA: Arachidonic acid
20-HETE: 20-Hydroxy-eicosatetraenoic acid
VGCCs: Voltage-gated calcium channels
ET1: Endothelin 1
IGF1: Insulin-like growth factor 1
MRs: Muscarinic ACh receptors
LPS: Lipopolysaccharide
TNF-α: Tumor necrosis factor-alpha
IL-1β: Interleukin-1 beta
MAPK: Mitogen-activated protein kinase
NF-κB: Nuclear factor kappa-light-chain-enhancer of activated B cells
IFN-γ: Interferon-gamma
CCLs: Chemokine ligands
MMPs: Matrix metalloproteinases
ROCK: Rho-associated protein kinase
VCAM-1: Vascular cell adhesion molecule 1
IgG: Immunoglobulin G
GM-CSF: Granulocyte–macrophage colony-stimulating factor
C1q: Component subunit 1q
A1: Reactive astrocytes
Mac-1: Macrophage-1 antigen
APOE4: Apolipoprotein E
CypA: Cyclophilin A
MCI: Mild cognitive impairment
p-tau: Phosphorylated tau
APOJ: Apolipoprotein J
ISF: Interstitial fluid
PCSK9: Proprotein convertase subtilisin/kexin type 9
BACE1: β-Site amyloid precursor protein cleaving enzyme 1
NOXs: NADPH oxidases
CAA: Cerebral amyloid angiopathy
eNOS: Endothelial nitric oxide synthase
LRP1-ICD: Intracellular domain of LRP1
αSyn: Alpha-synuclein preformed fibrils
PARP1: Poly (adenosine 5′-diphosphate-ribose) polymerase 1
proBDNF: Pro-brain-derived neurotrophic factor
6-OHDA: 6-Hydroxydopamine
COX-2: Cyclooxygenase-2
iNOS: Inducible nitric oxide synthase
MCP-1: Chemoattractant protein-1
OLs: Oligodendrocytes
EAE: Experimental autoimmune encephalomyelitis
ROS: Reactive oxygen and nitrogen species
NRs: NMDA receptors
APC: Activated Protein C
XQ-1H: 10-O-(N, N-dimethylaminoethyl) ginkgolide B methanesulfonate
iPSCs: Induced pluripotent stem cells
RvD: Resolvin D

## References

[1] Sweeney MD, Sagare AP, Zlokovic BV. Blood-brain barrier breakdown in Alzheimer disease and other neurodegenerative disorders. Nat Rev Neurol. 2018;14(3):133–50. 29377008 10.1038/nrneurol.2017.188 PMC5829048

[2] Iadecola C. The neurovascular unit coming of age: a journey through neurovascular coupling in health and disease. Neuron. 2017;96(1):17–42. 28957666 10.1016/j.neuron.2017.07.030 PMC5657612

[3] Vicario N, Parenti R. Connexins signatures of the neurovascular unit and their physio-pathological functions. Int J Mol Sci. 2022. 10.3390/ijms23179510. 36076908 10.3390/ijms23179510 PMC9455936

[4] Hartmann DA, Berthiaume AA, Grant RI, Harrill SA, Koski T, Tieu T, et al. Brain capillary pericytes exert a substantial but slow influence on blood flow. Nat Neurosci. 2021;24(5):633–45. 33603231 10.1038/s41593-020-00793-2 PMC8102366

[5] Berthiaume AA, Grant RI, McDowell KP, Underly RG, Hartmann DA, Levy M, et al. Dynamic remodeling of pericytes maintains capillary coverage in the adult mouse brain. Cell Rep. 2018;22(1):8–16. 29298435 10.1016/j.celrep.2017.12.016 PMC5782812

[6] Nikolakopoulou AM, Montagne A, Kisler K, Dai Z, Wang Y, Huuskonen MT, et al. Pericyte loss leads to circulatory failure and pleiotrophin depletion causing neuron loss. Nat Neurosci. 2019;22(7):1089–98. 31235908 10.1038/s41593-019-0434-z PMC6668719

[7] Kisler K, Nelson AR, Montagne A, Zlokovic BV. Cerebral blood flow regulation and neurovascular dysfunction in Alzheimer disease. Nat Rev Neurosci. 2017;18(7):419–34. 28515434 10.1038/nrn.2017.48 PMC5759779

[8] Sato Y, Falcone-Juengert J, Tominaga T, Su H, Liu J. Remodeling of the neurovascular unit following cerebral ischemia and hemorrhage. Cells. 2022. 10.3390/cells11182823. 36139398 10.3390/cells11182823 PMC9496956

[9] Ahmad A, Patel V, Xiao J, Khan MM. The role of neurovascular system in neurodegenerative diseases. Mol Neurobiol. 2020;57(11):4373–93. 32725516 10.1007/s12035-020-02023-z

[10] Xingi E, Koutsoudaki PN, Thanou I, Phan MS, Margariti M, Scheller A, et al. LPS-induced systemic inflammation affects the dynamic interactions of astrocytes and microglia with the vasculature of the mouse brain cortex. Cells. 2023. 10.3390/cells12101418. 37408252 10.3390/cells12101418 PMC10217139

[11] Sturtzel C. Endothelial cells. Adv Exp Med Biol. 2017;1003:71–91. 28667554 10.1007/978-3-319-57613-8_4

[12] Lochhead JJ, Yang JZ, Ronaldson PT, Davis TP. Structure, function, and regulation of the blood-brain barrier tight junction in central nervous system disorders. Front Physiol. 2020. 10.3389/fphys.2020.00914. 32848858 10.3389/fphys.2020.00914 PMC7424030

[13] Brightman MW, Reese TS. Junctions between intimately apposed cell membranes in the vertebrate brain. J Cell Biol. 1969;40(3):648–77. 5765759 10.1083/jcb.40.3.648 PMC2107650

[14] Westergaard E, Brightman MW. Transport of proteins across normal cerebral arterioles. J Comp Neurol. 1973;152(1):17–44. 4765853 10.1002/cne.901520103

[15] Butt AM, Jones HC, Abbott NJ. Electrical resistance across the blood-brain barrier in anaesthetized rats: a developmental study. J Physiol. 1990;429:47–62. 2277354 10.1113/jphysiol.1990.sp018243 PMC1181686

[16] Tietz S, Engelhardt B. Brain barriers: crosstalk between complex tight junctions and adherens junctions. J Cell Biol. 2015;209(4):493–506. 26008742 10.1083/jcb.201412147 PMC4442813

[17] Montagne A, Zhao Z, Zlokovic BV. Alzheimer’s disease: a matter of blood-brain barrier dysfunction? J Exp Med. 2017;214(11):3151–69. 29061693 10.1084/jem.20171406 PMC5679168

[18] Zhao Y, Gan L, Ren L, Lin Y, Ma C, Lin X. Factors influencing the blood-brain barrier permeability. Brain Res. 2022;1788: 147937. 35568085 10.1016/j.brainres.2022.147937

[19] Sweeney MD, Zhao Z, Montagne A, Nelson AR, Zlokovic BV. Blood-brain barrier: from physiology to disease and back. Physiol Rev. 2019;99(1):21–78. 30280653 10.1152/physrev.00050.2017 PMC6335099

[20] Hartsock A, Nelson WJ. Adherens and tight junctions: structure, function and connections to the actin cytoskeleton. Biochim Et Biophysica Acta Biomembr. 2008;1778(3):660–9. 10.1016/j.bbamem.2007.07.012 PMC2682436 17854762

[21] Abdullahi W, Tripathi D, Ronaldson PT. Blood-brain barrier dysfunction in ischemic stroke: targeting tight junctions and transporters for vascular protection. Am J Physiol Cell Physiol. 2018;315(3):C343–56. 29949404 10.1152/ajpcell.00095.2018 PMC6171039

[22] Pardridge WM. Drug transport across the blood-brain barrier. J Cereb Blood Flow Metab. 2012;32(11):1959–72. 22929442 10.1038/jcbfm.2012.126 PMC3494002

[23] Langen UH, Ayloo S, Gu C. Development and cell biology of the blood-brain barrier. Annu Rev Cell Dev Biol. 2019;35:591–613. 31299172 10.1146/annurev-cellbio-100617-062608 PMC8934576

[24] Knox EG, Aburto MR, Clarke G, Cryan JF, O’Driscoll CM. The blood-brain barrier in aging and neurodegeneration. Mol Psychiatry. 2022;27(6):2659–73. 35361905 10.1038/s41380-022-01511-z PMC9156404

[25] Teixeira MI, Lopes CM, Amaral MH, Costa PC. Current insights on lipid nanocarrier-assisted drug delivery in the treatment of neurodegenerative diseases. Eur J Pharm Biopharm. 2020;149:192–217. 31982574 10.1016/j.ejpb.2020.01.005

[26] Sweeney MD, Kisler K, Montagne A, Toga AW, Zlokovic BV. The role of brain vasculature in neurodegenerative disorders. Nat Neurosci. 2018;21(10):1318–31. 30250261 10.1038/s41593-018-0234-x PMC6198802

[27] Beyond the Amyloid Hypothesis of Alzheimer’s Disease. Tau pathology takes center stage. ACS Chem Neurosci. 2018;9(11):2519. 30458619 10.1021/acschemneuro.8b00610

[28] Jones AR, Shusta EV. Blood-brain barrier transport of therapeutics via receptor-mediation. Pharm Res. 2007;24(9):1759–71. 17619996 10.1007/s11095-007-9379-0 PMC2685177

[29] Pardridge WM. Drug and gene targeting to the brain with molecular trojan horses. Nat Rev Drug Discov. 2002;1(2):131–9. 12120094 10.1038/nrd725

[30] Miller DS. Regulation of ABC transporters blood-brain barrier: the good, the bad, and the ugly. Adv Cancer Res. 2015;125:43–70. 25640266 10.1016/bs.acr.2014.10.002

[31] Zhao Z, Nelson AR, Betsholtz C, Zlokovic BV. Establishment and dysfunction of the blood-brain barrier. Cell. 2015;163(5):1064–78. 26590417 10.1016/j.cell.2015.10.067 PMC4655822

[32] Hindle SJ, Munji RN, Dolghih E, Gaskins G, Orng S, Ishimoto H, et al. Evolutionarily conserved roles for blood-brain barrier xenobiotic transporters in endogenous steroid partitioning and behavior. Cell Rep. 2017;21(5):1304–16. 29091768 10.1016/j.celrep.2017.10.026 PMC5774027

[33] Langen UH, Ayloo S, Gu C. Development and cell biology of the blood-brain barrier. Annu Rev Cell Dev Biol. 2019;35:591–613. 31299172 10.1146/annurev-cellbio-100617-062608 PMC8934576

[34] Aird WC. Phenotypic heterogeneity of the endothelium: II representative vascular beds. Circ Res. 2007;100(2):174–90. 17272819 10.1161/01.RES.0000255690.03436.ae

[35] Daneman R, Zhou L, Agalliu D, Cahoy JD, Kaushal A, Barres BA. The mouse blood-brain barrier transcriptome: a new resource for understanding the development and function of brain endothelial cells. PLoS ONE. 2010;5(10): e13741. 21060791 10.1371/journal.pone.0013741 PMC2966423

[36] Achon Buil B, Tackenberg C, Rust R. Editing a gateway for cell therapy across the blood-brain barrier. Brain. 2023;146(3):823–41. 36397727 10.1093/brain/awac393 PMC9976985

[37] Zlokovic BV. Neurovascular pathways to neurodegeneration in Alzheimer’s disease and other disorders. Nat Rev Neurosci. 2011;12(12):723–38. 22048062 10.1038/nrn3114 PMC4036520

[38] Engelhardt B. Immune cell entry into the central nervous system: involvement of adhesion molecules and chemokines. J Neurol Sci. 2008;274(1–2):23–6. 18573502 10.1016/j.jns.2008.05.019

[39] Sweeney MD, Ayyadurai S, Zlokovic BV. Pericytes of the neurovascular unit: key functions and signaling pathways. Nat Neurosci. 2016;19(6):771–83. 27227366 10.1038/nn.4288 PMC5745011

[40] Yang AC, Vest RT, Kern F, Lee DP, Agam M, Maat CA, et al. A human brain vascular atlas reveals diverse mediators of Alzheimer’s risk. Nature. 2022;603(7903):885–92. 35165441 10.1038/s41586-021-04369-3 PMC9635042

[41] Profaci CP, Munji RN, Pulido RS, Daneman R. The blood-brain barrier in health and disease: important unanswered questions. J Exp Med. 2020. 10.1084/jem.20190062. 32211826 10.1084/jem.20190062 PMC7144528

[42] Winkler EA, Bell RD, Zlokovic BV. Central nervous system pericytes in health and disease. Nat Neurosci. 2011;14(11):1398–405. 22030551 10.1038/nn.2946 PMC4020628

[43] Armulik A, Genove G, Betsholtz C. Pericytes: developmental, physiological, and pathological perspectives, problems, and promises. Dev Cell. 2011;21(2):193–215. 21839917 10.1016/j.devcel.2011.07.001

[44] Daneman R, Zhou L, Kebede AA, Barres BA. Pericytes are required for blood-brain barrier integrity during embryogenesis. Nature. 2010;468(7323):562–6. 20944625 10.1038/nature09513 PMC3241506

[45] Sagare AP, Bell RD, Zhao Z, Ma Q, Winkler EA, Ramanathan A, et al. Pericyte loss influences Alzheimer-like neurodegeneration in mice. Nat Commun. 2013;4:2932. 24336108 10.1038/ncomms3932 PMC3945879

[46] Winkler EA, Sengillo JD, Sullivan JS, Henkel JS, Appel SH, Zlokovic BV. Blood-spinal cord barrier breakdown and pericyte reductions in amyotrophic lateral sclerosis. Acta Neuropathol. 2013;125(1):111–20. 22941226 10.1007/s00401-012-1039-8 PMC3535352

[47] Zheng Z, Chopp M, Chen J. Multifaceted roles of pericytes in central nervous system homeostasis and disease. J Cereb Blood Flow Metab. 2020;40(7):1381–401. 32208803 10.1177/0271678X20911331 PMC7308511

[48] Pandey K, Bessieres B, Sheng SL, Taranda J, Osten P, Sandovici I, et al. Neuronal activity drives IGF2 expression from pericytes to form long-term memory. Neuron. 2023. 10.1016/j.neuron.2023.08.030. 37788670 10.1016/j.neuron.2023.08.030 PMC10843759

[49] Diaz-Castro B, Robel S, Mishra A. Astrocyte endfeet in brain function and pathology: open questions. Annu Rev Neurosci. 2023;46:101–21. 36854317 10.1146/annurev-neuro-091922-031205

[50] Bardehle S, Kruger M, Buggenthin F, Schwausch J, Ninkovic J, Clevers H, et al. Live imaging of astrocyte responses to acute injury reveals selective juxtavascular proliferation. Nat Neurosci. 2013;16(5):580–6. 23542688 10.1038/nn.3371

[51] Yao Y, Chen ZL, Norris EH, Strickland S. Astrocytic laminin regulates pericyte differentiation and maintains blood brain barrier integrity. Nat Commun. 2014;5:3413. 24583950 10.1038/ncomms4413 PMC3992931

[52] Michinaga S, Koyama Y. Dual roles of astrocyte-derived factors in regulation of blood-brain barrier function after brain damage. Int J Mol Sci. 2019. 10.3390/ijms20030571. 30699952 10.3390/ijms20030571 PMC6387062

[53] Heithoff BP, George KK, Phares AN, Zuidhoek IA, Munoz-Ballester C, Robel S. Astrocytes are necessary for blood-brain barrier maintenance in the adult mouse brain. Glia. 2021;69(2):436–72. 32955153 10.1002/glia.23908 PMC7736206

[54] Attwell D, Buchan AM, Charpak S, Lauritzen M, Macvicar BA, Newman EA. Glial and neuronal control of brain blood flow. Nature. 2010;468(7321):232–43. 21068832 10.1038/nature09613 PMC3206737

[55] Cohen-Salmon M, Slaoui L, Mazare N, Gilbert A, Oudart M, Alvear-Perez R, et al. Astrocytes in the regulation of cerebrovascular functions. Glia. 2021;69(4):817–41. 33058289 10.1002/glia.23924

[56] Zhou B, Zuo YX, Jiang RT. Astrocyte morphology: diversity, plasticity, and role in neurological diseases. CNS Neurosci Ther. 2019;25(6):665–73. 30929313 10.1111/cns.13123 PMC6515705

[57] Trillo-Contreras JL, Ramirez-Lorca R, Villadiego J, Echevarria M. Cellular distribution of brain aquaporins and their contribution to cerebrospinal fluid homeostasis and hydrocephalus. Biomolecules. 2022. 10.3390/biom12040530. 35454119 10.3390/biom12040530 PMC9025855

[58] Iliff JJ, Wang M, Liao Y, Plogg BA, Peng W, Gundersen GA, et al. A paravascular pathway facilitates CSF flow through the brain parenchyma and the clearance of interstitial solutes, including amyloid beta. Sci Transl Med. 2012. 10.1126/scitranslmed.3003748. 22896675 10.1126/scitranslmed.3003748 PMC3551275

[59] Aldea R, Weller RO, Wilcock DM, Carare RO, Richardson G. Cerebrovascular smooth muscle cells as the drivers of intramural periarterial drainage of the brain. Front Aging Neurosci. 2019;11:1. 30740048 10.3389/fnagi.2019.00001 PMC6357927

[60] Diem AK, Carare RO, Weller RO, Bressloff NW. A control mechanism for intra-mural peri-arterial drainage via astrocytes: How neuronal activity could improve waste clearance from the brain. PLoS ONE. 2018;13(10): e0205276. 30286191 10.1371/journal.pone.0205276 PMC6171921

[61] Xue X, Zhang W, Zhu J, Chen X, Zhou S, Xu Z, et al. Aquaporin-4 deficiency reduces TGF-beta1 in mouse midbrains and exacerbates pathology in experimental Parkinson’s disease. J Cell Mol Med. 2019;23(4):2568–82. 30680924 10.1111/jcmm.14147 PMC6433854

[62] Morris AW, Sharp MM, Albargothy NJ, Fernandes R, Hawkes CA, Verma A, et al. Vascular basement membranes as pathways for the passage of fluid into and out of the brain. Acta Neuropathol. 2016;131(5):725–36. 26975356 10.1007/s00401-016-1555-z PMC4835509

[63] Thomsen MS, Routhe LJ, Moos T. The vascular basement membrane in the healthy and pathological brain. J Cereb Blood Flow Metab. 2017;37(10):3300–17. 28753105 10.1177/0271678X17722436 PMC5624399

[64] Ceafalan LC, Fertig TE, Gheorghe TC, Hinescu ME, Popescu BO, Pahnke J, et al. Age-related ultrastructural changes of the basement membrane in the mouse blood-brain barrier. J Cell Mol Med. 2019;23(2):819–27. 30450815 10.1111/jcmm.13980 PMC6349169

[65] Thomsen MS, Birkelund S, Burkhart A, Stensballe A, Moos T. Synthesis and deposition of basement membrane proteins by primary brain capillary endothelial cells in a murine model of the blood-brain barrier. J Neurochem. 2017;140(5):741–54. 27456748 10.1111/jnc.13747

[66] Gautam J, Zhang X, Yao Y. The role of pericytic laminin in blood brain barrier integrity maintenance. Sci Rep. 2016;6:36450. 27808256 10.1038/srep36450 PMC5093438

[67] Menezes MJ, McClenahan FK, Leiton CV, Aranmolate A, Shan X, Colognato H. The extracellular matrix protein laminin alpha2 regulates the maturation and function of the blood-brain barrier. J Neurosci. 2014;34(46):15260–80. 25392494 10.1523/JNEUROSCI.3678-13.2014 PMC6608454

[68] Kenne E, Soehnlein O, Genove G, Rotzius P, Eriksson EE, Lindbom L. Immune cell recruitment to inflammatory loci is impaired in mice deficient in basement membrane protein laminin alpha4. J Leukoc Biol. 2010;88(3):523–8. 20483922 10.1189/jlb.0110043

[69] Poschl E, Schlotzer-Schrehardt U, Brachvogel B, Saito K, Ninomiya Y, Mayer U. Collagen IV is essential for basement membrane stability but dispensable for initiation of its assembly during early development. Development. 2004;131(7):1619–28. 14998921 10.1242/dev.01037

[70] Nakamura K, Ikeuchi T, Nara K, Rhodes CS, Zhang P, Chiba Y, et al. Perlecan regulates pericyte dynamics in the maintenance and repair of the blood–brain barrier. J Cell Biol. 2019;218(10):3506–25. 31541017 10.1083/jcb.201807178 PMC6781430

[71] Trout AL, Rutkai I, Biose IJ, Bix GJ. Review of alterations in perlecan-associated vascular risk factors in dementia. Int J Mol Sci. 2020. 10.3390/ijms21020679. 31968632 10.3390/ijms21020679 PMC7013765

[72] Lord MS, Chuang CY, Melrose J, Davies MJ, Iozzo RV, Whitelock JM. The role of vascular-derived perlecan in modulating cell adhesion, proliferation and growth factor signaling. Matrix Biol. 2014;35:112–22. 24509440 10.1016/j.matbio.2014.01.016 PMC5030467

[73] Tallquist MD, French WJ, Soriano P. Additive effects of PDGF receptor beta signaling pathways in vascular smooth muscle cell development. PLoS Biol. 2003;1(2):E52. 14624252 10.1371/journal.pbio.0000052 PMC261889

[74] Bell RD, Winkler EA, Sagare AP, Singh I, LaRue B, Deane R, et al. Pericytes control key neurovascular functions and neuronal phenotype in the adult brain and during brain aging. Neuron. 2010;68(3):409–27. 21040844 10.1016/j.neuron.2010.09.043 PMC3056408

[75] Franco M, Roswall P, Cortez E, Hanahan D, Pietras K. Pericytes promote endothelial cell survival through induction of autocrine VEGF-A signaling and Bcl-w expression. Blood. 2011;118(10):2906–17. 21778339 10.1182/blood-2011-01-331694 PMC3172806

[76] Schoch HJ, Fischer S, Marti HH. Hypoxia-induced vascular endothelial growth factor expression causes vascular leakage in the brain. Brain. 2002;125(Pt 11):2549–57. 12390979 10.1093/brain/awf257

[77] Argaw AT, Gurfein BT, Zhang Y, Zameer A, John GR. VEGF-mediated disruption of endothelial CLN-5 promotes blood-brain barrier breakdown. Proc Natl Acad Sci U S A. 2009;106(6):1977–82. 19174516 10.1073/pnas.0808698106 PMC2644149

[78] Reyahi A, Nik AM, Ghiami M, Gritli-Linde A, Ponten F, Johansson BR, et al. Foxf2 is required for brain pericyte differentiation and development and maintenance of the blood-brain barrier. Dev Cell. 2015;34(1):19–32. 26120030 10.1016/j.devcel.2015.05.008

[79] Darland DC, D’Amore PA. TGF beta is required for the formation of capillary-like structures in three-dimensional cocultures of 10T1/2 and endothelial cells. Angiogenesis. 2001;4(1):11–20. 11824373 10.1023/A:1016611824696

[80] Andreone BJ, Chow BW, Tata A, Lacoste B, Ben-Zvi A, Bullock K, et al. Blood-brain barrier permeability is regulated by lipid transport-dependent suppression of caveolae-mediated transcytosis. Neuron. 2017. 10.1016/j.neuron.2017.03.043. 28416077 10.1016/j.neuron.2017.03.043 PMC5474951

[81] Qu C, Song H, Shen J, Xu L, Li Y, Qu C, et al. Mfsd2a reverses spatial learning and memory impairment caused by chronic cerebral hypoperfusion via protection of the blood-brain barrier. Front Neurosci. 2020. 10.3389/fnins.2020.00461. 32612494 10.3389/fnins.2020.00461 PMC7308492

[82] Dietrich JB. The adhesion molecule ICAM-1 and its regulation in relation with the blood-brain barrier. J Neuroimmunol. 2002;128(1–2):58–68. 12098511 10.1016/S0165-5728(02)00114-5

[83] Eilam R, Segal M, Malach R, Sela M, Arnon R, Aharoni R. Astrocyte disruption of neurovascular communication is linked to cortical damage in an animal model of multiple sclerosis. Glia. 2018;66(5):1098–117. 29424049 10.1002/glia.23304

[84] Haruwaka K, Ikegami A, Tachibana Y, Ohno N, Konishi H, Hashimoto A, et al. Dual microglia effects on blood brain barrier permeability induced by systemic inflammation. Nat Commun. 2019;10(1):5816. 31862977 10.1038/s41467-019-13812-z PMC6925219

[85] Yu X, Ji C, Shao A. Neurovascular unit dysfunction and neurodegenerative disorders. Front Neurosci. 2020;14:334. 32410936 10.3389/fnins.2020.00334 PMC7201055

[86] Attwell D, Laughlin SB. An energy budget for signaling in the grey matter of the brain. J Cereb Blood Flow Metab. 2001;21(10):1133–45. 11598490 10.1097/00004647-200110000-00001

[87] Horlyck S, Cai C, Helms HCC, Lauritzen M, Brodin B. ATP induces contraction of cultured brain capillary pericytes via activation of P2Y-type purinergic receptors. Am J Physiol Heart Circ Physiol. 2021;320(2):H699–712. 33306443 10.1152/ajpheart.00560.2020

[88] Ko KR, Ngai AC, Winn HR. Role of adenosine in regulation of regional cerebral blood flow in sensory cortex. Am J Physiol. 1990;259(6 Pt 2):H1703-1708. 2260697 10.1152/ajpheart.1990.259.6.H1703

[89] Jackson WF. Tuning the signal: ATP-sensitive K (+) channels direct blood flow to cerebral capillaries. Sci Signal. 2022;15(727):eabo1118. 35349301 10.1126/scisignal.abo1118

[90] Peppiatt CM, Howarth C, Mobbs P, Attwell D. Bidirectional control of CNS capillary diameter by pericytes. Nature. 2006;443(7112):700–4. 17036005 10.1038/nature05193 PMC1761848

[91] Tota S, Goel R, Pachauri SD, Rajasekar N, Najmi AK, Hanif K, et al. Effect of angiotensin II on spatial memory, cerebral blood flow, cholinergic neurotransmission, and brain derived neurotrophic factor in rats. Psychopharmacology. 2013;226(2):357–69. 23192311 10.1007/s00213-012-2913-8

[92] Lecrux C, Hamel E. Neuronal networks and mediators of cortical neurovascular coupling responses in normal and altered brain states. Philos Trans R Soc Lond B Biol Sci. 2016. 10.1098/rstb.2015.0350. 27574304 10.1098/rstb.2015.0350 PMC5003852

[93] Hamilton NB, Attwell D, Hall CN. Pericyte-mediated regulation of capillary diameter: a component of neurovascular coupling in health and disease. Front Neuroenergetics. 2010. 10.3389/fnene.2010.00005. 20725515 10.3389/fnene.2010.00005 PMC2912025

[94] Hall CN, Reynell C, Gesslein B, Hamilton NB, Mishra A, Sutherland BA, et al. Capillary pericytes regulate cerebral blood flow in health and disease. Nature. 2014;508(7494):55–60. 24670647 10.1038/nature13165 PMC3976267

[95] Kitchen P, Salman MM, Halsey AM, Clarke-Bland C, MacDonald JA, Ishida H, et al. Targeting aquaporin-4 subcellular localization to treat central nervous system edema. Cell. 2020. 10.1016/j.cell.2020.03.037. 32413299 10.1016/j.cell.2020.03.037 PMC7242911

[96] Stokum JA, Kurland DB, Gerzanich V, Simard JM. Mechanisms of astrocyte-mediated cerebral edema. Neurochem Res. 2015;40(2):317–28. 24996934 10.1007/s11064-014-1374-3 PMC4284155

[97] Patabendige A, Singh A, Jenkins S, Sen J, Chen R. Astrocyte activation in neurovascular damage and repair following ischaemic stroke. Int J Mol Sci. 2021. 10.3390/ijms22084280. 33924191 10.3390/ijms22084280 PMC8074612

[98] Bordoni L, Thoren AE, Gutierrez-Jimenez E, Abjorsbraten KS, Bjornstad DM, Tang W, et al. Deletion of aquaporin-4 improves capillary blood flow distribution in brain edema. Glia. 2023;71(11):2559–72. 37439315 10.1002/glia.24439 PMC10952478

[99] Nortley R, Korte N, Izquierdo P, Hirunpattarasilp C, Mishra A, Jaunmuktane Z, et al. Amyloid beta oligomers constrict human capillaries in Alzheimer’s disease via signaling to pericytes. Science. 2019. 10.1126/science.aav9518. 31221773 10.1126/science.aav9518 PMC6658218

[100] Kisler K, Nelson AR, Rege SV, Ramanathan A, Wang Y, Ahuja A, et al. Pericyte degeneration leads to neurovascular uncoupling and limits oxygen supply to brain. Nat Neurosci. 2017;20(3):406–16. 28135240 10.1038/nn.4489 PMC5323291

[101] Nikolakopoulou AM, Montagne A, Kisler K, Dai Z, Wang Y, Huuskonen MT, et al. Pericyte loss leads to circulatory failure and pleiotrophin depletion causing neuron loss. Nat Neurosci. 2019;22(7):1089–98. 31235908 10.1038/s41593-019-0434-z PMC6668719

[102] Hirunpattarasilp C, Attwell D, Freitas F. The role of pericytes in brain disorders: from the periphery to the brain. J Neurochem. 2019;150(6):648–65. 31106417 10.1111/jnc.14725

[103] Mishra A, Reynolds JP, Chen Y, Gourine AV, Rusakov DA, Attwell D. Astrocytes mediate neurovascular signaling to capillary pericytes but not to arterioles. Nat Neurosci. 2016;19(12):1619–27. 27775719 10.1038/nn.4428 PMC5131849

[104] Zhou SY, Guo ZN, Zhang DH, Qu Y, Jin H. The role of pericytes in ischemic stroke: fom cellular functions to therapeutic targets. Front Mol Neurosci. 2022;15: 866700. 35493333 10.3389/fnmol.2022.866700 PMC9043812

[105] Carmeliet P, Ferreira V, Breier G, Pollefeyt S, Kieckens L, Gertsenstein M, et al. Abnormal blood vessel development and lethality in embryos lacking a single VEGF allele. Nature. 1996;380(6573):435–9. 8602241 10.1038/380435a0

[106] Longden TA, Dabertrand F, Koide M, Gonzales AL, Tykocki NR, Brayden JE, et al. Capillary K(+)-sensing initiates retrograde hyperpolarization to increase local cerebral blood flow. Nat Neurosci. 2017;20(5):717–26. 28319610 10.1038/nn.4533 PMC5404963

[107] Szutowicz A, Bielarczyk H, Ronowska A, Gul-Hinc S, Klimaszewska-Tata J, Dys A, et al. Intracellular redistribution of acetyl-CoA, the pivotal point in differential susceptibility of cholinergic neurons and glial cells to neurodegenerative signals. Biochem Soc T. 2014;42:1101–6. 10.1042/BST20140078 25110009

[108] Garcia-Bonilla L, Brea D, Benakis C, Lane DA, Murphy M, Moore J, et al. Endogenous protection from ischemic brain injury by preconditioned monocytes. J Neurosci. 2018;38(30):6722–36. 29946039 10.1523/JNEUROSCI.0324-18.2018 PMC6067076

[109] Quaranta DV, Weaver RR, Baumann KK, Fujimoto T, Williams LM, Kim HC, et al. Transport of the proinflammatory chemokines C-C motif chemokine ligand 2 (MCP-1) and C-C motif chemokine ligand 5 (RANTES) across the intact mouse blood-brain barrier is inhibited by heparin and eprodisate and increased with systemic inflammation. J Pharmacol Exp Ther. 2023;384(1):205–23. 36310035 10.1124/jpet.122.001380 PMC9827507

[110] Banks WA, Robinson SM. Minimal penetration of lipopolysaccharide across the murine blood-brain barrier. Brain Behav Immun. 2010;24(1):102–9. 19735725 10.1016/j.bbi.2009.09.001 PMC2789209

[111] Herkenham M, Lee HY, Baker RA. Temporal and spatial patterns ofc-fos mRNA induced by intravenous interleukin-1: a cascade of non-neuronal cellular activation at the blood-brain barrier. J Comp Neurol. 1998;400(2):175–96. 9766398 10.1002/(SICI)1096-9861(19981019)400:2<175::AID-CNE2>3.0.CO;2-6

[112] Quan N, He L, Lai W. Endothelial activation is an intermediate step for peripheral lipopolysaccharide induced activation of paraventricular nucleus. Brain Res Bull. 2003;59(6):447–52. 12576141 10.1016/S0361-9230(02)00951-6

[113] Wilhelms DB, Kirilov M, Mirrasekhian E, Eskilsson A, Kugelberg UO, Klar C, et al. Deletion of prostaglandin E2 synthesizing enzymes in brain endothelial cells attenuates inflammatory fever. J Neurosci. 2014;34(35):11684–90. 25164664 10.1523/JNEUROSCI.1838-14.2014 PMC6608410

[114] Larochelle C, Alvarez JI, Prat A. How do immune cells overcome the blood-brain barrier in multiple sclerosis? FEBS Lett. 2011;585(23):3770–80. 21550344 10.1016/j.febslet.2011.04.066

[115] Labus J, Woltje K, Stolte KN, Hackel S, Kim KS, Hildmann A, et al. IL-1beta promotes transendothelial migration of PBMCs by upregulation of the FN/alpha(5)beta(1) signalling pathway in immortalised human brain microvascular endothelial cells. Exp Cell Res. 2018;373(1–2):99–111. 30342992 10.1016/j.yexcr.2018.10.002

[116] Tan S, Shan Y, Lin Y, Liao S, Zhang B, Zeng Q, et al. Neutralization of interleukin-9 ameliorates experimental stroke by repairing the blood-brain barrier via down-regulation of astrocyte-derived vascular endothelial growth factor-A. FASEB J. 2019;33(3):4376–87. 30694693 10.1096/fj.201801595RR

[117] Labus J, Hackel S, Lucka L, Danker K. Interleukin-1beta induces an inflammatory response and the breakdown of the endothelial cell layer in an improved human THBMEC-based in vitro blood-brain barrier model. J Neurosci Method. 2014;228:35–45. 10.1016/j.jneumeth.2014.03.002 24631939

[118] Menard C, Pfau ML, Hodes GE, Kana V, Wang VX, Bouchard S, et al. Social stress induces neurovascular pathology promoting depression. Nat Neurosci. 2017;20(12):1752–60. 29184215 10.1038/s41593-017-0010-3 PMC5726568

[119] Chen W, Ju XZ, Lu Y, Ding XW, Miao CH, Chen JW. Propofol improved hypoxia-impaired integrity of blood-brain barrier via modulating the expression and phosphorylation of zonula occludens-1. CNS Neurosci Ther. 2019;25(6):704–13. 30680941 10.1111/cns.13101 PMC6515893

[120] Wong D, Dorovini-Zis K, Vincent SR. Cytokines, nitric oxide, and cGMP modulate the permeability of an in vitro model of the human blood-brain barrier. Exp Neurol. 2004;190(2):446–55. 15530883 10.1016/j.expneurol.2004.08.008

[121] Qin LH, Huang W, Mo XA, Chen YL, Wu XH. LPS induces occludin dysregulation in cerebral microvascular endothelial cells via MAPK signaling and augmenting MMP-2 levels. Oxid Med Cell Longev. 2015;2015: 120641. 26290681 10.1155/2015/120641 PMC4531183

[122] Han D, Fang W, Zhang R, Wei J, Kodithuwakku ND, Sha L, et al. Clematichinenoside protects blood brain barrier against ischemic stroke superimposed on systemic inflammatory challenges through up-regulating A20. Brain Behav Immun. 2016;51:56–69. 26231971 10.1016/j.bbi.2015.07.025

[123] Liu X, Sui B, Sun J. Blood-brain barrier dysfunction induced by silica NPs in vitro and in vivo: Involvement of oxidative stress and Rho-kinase/JNK signaling pathways. Biomaterials. 2017;121:64–82. 28081460 10.1016/j.biomaterials.2017.01.006

[124] Gao M, Lu W, Shu Y, Yang Z, Sun S, Xu J, et al. Poldip2 mediates blood-brain barrier disruption and cerebral edema by inducing AQP4 polarity loss in mouse bacterial meningitis model. CNS Neurosci Ther. 2020;26(12):1288–302. 32790044 10.1111/cns.13446 PMC7702237

[125] Huang X, Hussain B, Chang J. Peripheral inflammation and blood-brain barrier disruption: effects and mechanisms. CNS Neurosci Ther. 2021;27(1):36–47. 33381913 10.1111/cns.13569 PMC7804893

[126] Nakamuta S, Endo H, Higashi Y, Kousaka A, Yamada H, Yano M, et al. Human immunodeficiency virus type 1 gp120-mediated disruption of tight junction proteins by induction of proteasome-mediated degradation of zonula occludens-1 and -2 in human brain microvascular endothelial cells. J Neurovirol. 2008;14(3):186–95. 18569453 10.1080/13550280801993630

[127] Yu CC, Chen HL, Chen MH, Lu CH, Tsai NW, Huang CC, et al. Vascular inflammation is a risk factor associated with brain atrophy and disease severity in Parkinson’s disease: a case-control study. Oxid Med Cell Longev. 2020;2020:2591248. 32733633 10.1155/2020/2591248 PMC7376437

[128] Yousef H, Czupalla CJ, Lee D, Chen MB, Burke AN, Zera KA, et al. Aged blood impairs hippocampal neural precursor activity and activates microglia via brain endothelial cell VCAM1. Nat Med. 2019;25(6):988–1000. 31086348 10.1038/s41591-019-0440-4 PMC6642642

[129] Souza PS, Goncalves ED, Pedroso GS, Farias HR, Junqueira SC, Marcon R, et al. Physical exercise attenuates experimental autoimmune encephalomyelitis by inhibiting peripheral immune response and blood-brain barrier disruption. Mol Neurobiol. 2017;54(6):4723–37. 27447807 10.1007/s12035-016-0014-0

[130] Mendiola AS, Yan Z, Dixit K, Johnson JR, Bouhaddou M, Meyer-Franke A, et al. Defining blood-induced microglia functions in neurodegeneration through multiomic profiling. Nat Immunol. 2023;24(7):1173–87. 37291385 10.1038/s41590-023-01522-0 PMC10307624

[131] Blecharz-Lang KG, Wagner J, Fries A, Nieminen-Kelhä M, Rösner J, Schneider UC, et al. Interleukin 6-mediated endothelial barrier disturbances can be attenuated by blockade of the il6 receptor expressed in brain microvascular endothelial cells. Transl Stroke Res. 2018;9(6):631–42. 29429002 10.1007/s12975-018-0614-2

[132] Liddelow SA, Barres BA. Reactive astrocytes: production, function, and therapeutic potential. Immunity. 2017;46(6):957–67. 28636962 10.1016/j.immuni.2017.06.006

[133] Kant R, Halder SK, Fernandez JA, Griffin JH, Milner R. Activated protein C attenuates experimental autoimmune encephalomyelitis progression by enhancing vascular integrity and suppressing microglial activation. Front Neurosci. 2020;14:333. 32351356 10.3389/fnins.2020.00333 PMC7174764

[134] Jolivel V, Bicker F, Biname F, Ploen R, Keller S, Gollan R, et al. Perivascular microglia promote blood vessel disintegration in the ischemic penumbra. Acta Neuropathol. 2015;129(2):279–95. 25500713 10.1007/s00401-014-1372-1

[135] van Horssen J, Vos CM, Admiraal L, van Haastert ES, Montagne L, van der Valk P, et al. Matrix metalloproteinase-19 is highly expressed in active multiple sclerosis lesions. Neuropathol Appl Neurobiol. 2006;32(6):585–93. 17083473 10.1111/j.1365-2990.2006.00766.x

[136] Pieper C, Marek JJ, Unterberg M, Schwerdtle T, Galla HJ. Brain capillary pericytes contribute to the immune defense in response to cytokines or LPS in vitro. Brain Res. 2014;1550:1–8. 24418464 10.1016/j.brainres.2014.01.004

[137] Nation DA, Sweeney MD, Montagne A, Sagare AP, D’Orazio LM, Pachicano M, et al. Blood-brain barrier breakdown is an early biomarker of human cognitive dysfunction. Nat Med. 2019;25(2):270–6. 30643288 10.1038/s41591-018-0297-y PMC6367058

[138] Iturria-Medina Y, Sotero RC, Toussaint PJ, Mateos-Perez JM, Evans AC. Early role of vascular dysregulation on late-onset Alzheimer’s disease based on multifactorial data-driven analysis. Nat Commun. 2016. 10.1038/ncomms11934. 27327500 10.1038/ncomms11934 PMC4919512

[139] Cullen KM, Kocsi Z, Stone J. Pericapillary haem-rich deposits: evidence for microhaemorrhages in aging human cerebral cortex. J Cereb Blood Flow Metab. 2005;25(12):1656–67. 15917745 10.1038/sj.jcbfm.9600155

[140] Fiala M, Liu QN, Sayre J, Pop V, Brahmandam V, Graves MC, et al. Cyclooxygenase-2-positive macrophages infiltrate the Alzheimer’s disease brain and damage the blood-brain barrier. Eur J Clin Invest. 2002;32(5):360–71. 12027877 10.1046/j.1365-2362.2002.00994.x

[141] Zenaro E, Pietronigro E, Della Bianca V, Piacentino G, Marongiu L, Budui S, et al. Neutrophils promote Alzheimer’s disease-like pathology and cognitive decline via LFA-1 integrin. Nat Med. 2015;21(8):880–6. 26214837 10.1038/nm.3913

[142] Cortes-Canteli M, Paul J, Norris EH, Bronstein R, Ahn HJ, Zamolodchikov D, et al. Fibrinogen and beta-amyloid association alters thrombosis and fibrinolysis: a possible contributing factor to Alzheimer’s disease. Neuron. 2010;66(5):695–709. 20547128 10.1016/j.neuron.2010.05.014 PMC2895773

[143] Hultman K, Strickland S, Norris EH. The APOE varepsilon4/varepsilon4 genotype potentiates vascular fibrin (ogen) deposition in amyloid-laden vessels in the brains of Alzheimer’s disease patients. J Cereb Blood Flow Metab. 2013;33(8):1251–8. 23652625 10.1038/jcbfm.2013.76 PMC3734776

[144] Zipser BD, Johanson CE, Gonzalez L, Berzin TM, Tavares R, Hulette CM, et al. Microvascular injury and blood-brain barrier leakage in Alzheimer’s disease. Neurobiol Aging. 2007;28(7):977–86. 16782234 10.1016/j.neurobiolaging.2006.05.016

[145] Inoue Y, Ueda M, Masuda T, Misumi Y, Yamashita T, Ando Y. Memantine, a noncompetitive N-methyl-D-aspartate receptor antagonist, attenuates cerebral amyloid angiopathy by increasing insulin-degrading enzyme expression. Mol Neurobiol. 2019;56(12):8573–88. 31280448 10.1007/s12035-019-01678-7

[146] Montagne A, Barnes SR, Sweeney MD, Halliday MR, Sagare AP, Zhao Z, et al. Blood-brain barrier breakdown in the aging human hippocampus. Neuron. 2015;85(2):296–302. 25611508 10.1016/j.neuron.2014.12.032 PMC4350773

[147] van de Haar HJ, Jansen JFA, van Osch MJP, van Buchem MA, Muller M, Wong SM, et al. Neurovascular unit impairment in early Alzheimer’s disease measured with magnetic resonance imaging. Neurobiol Aging. 2016;45:190–6. 27459939 10.1016/j.neurobiolaging.2016.06.006

[148] van de Haar HJ, Burgmans S, Jansen JF, van Osch MJ, van Buchem MA, Muller M, et al. Blood-brain barrier leakage in patients with early Alzheimer disease. Radiology. 2016;281(2):527–35. 27243267 10.1148/radiol.2016152244

[149] van de Haar HJ, Jansen JFA, Jeukens C, Burgmans S, van Buchem MA, Muller M, et al. Subtle blood-brain barrier leakage rate and spatial extent: considerations for dynamic contrast-enhanced MRI. Med Phys. 2017;44(8):4112–25. 28493613 10.1002/mp.12328

[150] Montagne A, Nation DA, Sagare AP, Barisano G, Sweeney MD, Chakhoyan A, et al. APOE4 leads to blood-brain barrier dysfunction predicting cognitive decline. Nature. 2020;581(7806):71–6. 32376954 10.1038/s41586-020-2247-3 PMC7250000

[151] Halliday MR, Rege SV, Ma Q, Zhao Z, Miller CA, Winkler EA, et al. Accelerated pericyte degeneration and blood-brain barrier breakdown in apolipoprotein E4 carriers with Alzheimer’s disease. J Cereb Blood Flow Metab. 2016;36(1):216–27. 25757756 10.1038/jcbfm.2015.44 PMC4758554

[152] Montagne A, Nikolakopoulou AM, Huuskonen MT, Sagare AP, Lawson EJ, Lazic D, et al. APOE4 accelerates advanced-stage vascular and neurodegenerative disorder in old Alzheimer’s mice via cyclophilin a independently of amyloid-beta. Nat Aging. 2021;1(6):506–20. 35291561 10.1038/s43587-021-00073-z PMC8920485

[153] Salloway S, Gur T, Berzin T, Tavares R, Zipser B, Correia S, et al. Effect of APOE genotype on microvascular basement membrane in Alzheimer’s disease. J Neurol Sci. 2002;203–204:183–7. 12417381 10.1016/S0022-510X(02)00288-5

[154] Chen Z, Zhong C. Decoding Alzheimer’s disease from perturbed cerebral glucose metabolism: implications for diagnostic and therapeutic strategies. Prog Neurobiol. 2013;108:21–43. 23850509 10.1016/j.pneurobio.2013.06.004

[155] Minoshima S, Giordani B, Berent S, Frey KA, Foster NL, Kuhl DE. Metabolic reduction in the posterior cingulate cortex in very early Alzheimer’s disease. Ann Neurol. 1997;42(1):85–94. 9225689 10.1002/ana.410420114

[156] An Y, Varma VR, Varma S, Casanova R, Dammer E, Pletnikova O, et al. Evidence for brain glucose dysregulation in Alzheimer’s disease. Alzheimers Dement. 2018;14(3):318–29. 29055815 10.1016/j.jalz.2017.09.011 PMC5866736

[157] Chen Y, Wolk DA, Reddin JS, Korczykowski M, Martinez PM, Musiek ES, et al. Voxel-level comparison of arterial spin-labeled perfusion MRI and FDG-PET in Alzheimer disease. Neurology. 2011;77(22):1977–85. 22094481 10.1212/WNL.0b013e31823a0ef7 PMC3235355

[158] Gil-Iturbe E, Solas M, Cuadrado-Tejedo M, Garcia-Osta A, Escote X, Ramirez MJ, et al. GLUT12 expression in brain of mouse models of Alzheimer’s disease. Mol Neurobiol. 2020;57(2):798–805. 31473905 10.1007/s12035-019-01743-1

[159] Sperling RA, Aisen PS, Beckett LA, Bennett DA, Craft S, Fagan AM, et al. Toward defining the preclinical stages of Alzheimer’s disease: recommendations from the national institute on aging-Alzheimer’s association workgroups on diagnostic guidelines for Alzheimer’s disease. Alzheimers Dement. 2011;7(3):280–92. 21514248 10.1016/j.jalz.2011.03.003 PMC3220946

[160] Martin-Macintosh EL, Broski SM, Johnson GB, Hunt CH, Cullen EL, Peller PJ. Multimodality imaging of neurodegenerative processes: part 1, the basics and common dementias. Am J Roentgenol. 2016;207(4):871–82. 27505704 10.2214/AJR.14.12842

[161] Bailly M, Destrieux C, Hommet C, Mondon K, Cottier JP, Beaufils E, et al. Precuneus and cingulate cortex atrophy and hypometabolism in patients with Alzheimer’s disease and mild cognitive impairment: MRI and (18)F-FDG PET quantitative analysis using freesurfer. Biomed Res Int. 2015;2015: 583931. 26346648 10.1155/2015/583931 PMC4539420

[162] Landau SM, Harvey D, Madison CM, Koeppe RA, Reiman EM, Foster NL, et al. Associations between cognitive, functional, and FDG-PET measures of decline in AD and MCI. Neurobiol Aging. 2011;32(7):1207–18. 19660834 10.1016/j.neurobiolaging.2009.07.002 PMC2891865

[163] Winkler EA, Nishida Y, Sagare AP, Rege SV, Bell RD, Perlmutter D, et al. GLUT1 reductions exacerbate Alzheimer’s disease vasculo-neuronal dysfunction and degeneration. Nat Neurosci. 2015;18(4):521–30. 25730668 10.1038/nn.3966 PMC4734893

[164] Taccola C, Barneoud P, Cartot-Cotton S, Valente D, Schussler N, Saubamea B, et al. Modifications of physical and functional integrity of the blood-brain barrier in an inducible mouse model of neurodegeneration. Neuropharmacology. 2021;191: 108588. 33940010 10.1016/j.neuropharm.2021.108588

[165] Majerova P, Michalicova A, Cente M, Hanes J, Vegh J, Kittel A, et al. Trafficking of immune cells across the blood-brain barrier is modulated by neurofibrillary pathology in tauopathies. PLoS ONE. 2019;14(5): e0217216. 31120951 10.1371/journal.pone.0217216 PMC6532920

[166] Storck SE, Pietrzik CU. Endothelial LRP1—a potential target for the treatment of Alzheimer’s disease : theme: drug discovery, development and delivery in Alzheimer’s disease guest editor: davide brambilla. Pharm Res. 2017;34(12):2637–51. 28948494 10.1007/s11095-017-2267-3

[167] Bell RD, Sagare AP, Friedman AE, Bedi GS, Holtzman DM, Deane R, et al. Transport pathways for clearance of human Alzheimer’s amyloid beta-peptide and apolipoproteins E and J in the mouse central nervous system. J Cereb Blood Flow Metab. 2007;27(5):909–18. 17077814 10.1038/sj.jcbfm.9600419 PMC2853021

[168] Chai AB, Hartz AMS, Gao X, Yang A, Callaghan R, Gelissen IC. New evidence for P-gp-mediated export of amyloid-β peptides in molecular, blood-brain barrier and neuronal models. Int J Mol Sci. 2020;22(1):246. 33383667 10.3390/ijms22010246 PMC7795149

[169] Chiu C, Miller MC, Monahan R, Osgood DP, Stopa EG, Silverberg GD. P-glycoprotein expression and amyloid accumulation in human aging and Alzheimer’s disease: preliminary observations. Neurobiol Aging. 2015;36(9):2475–82. 26159621 10.1016/j.neurobiolaging.2015.05.020

[170] Park R, Kook SY, Park JC, Mook-Jung I. Abeta1-42 reduces P-glycoprotein in the blood-brain barrier through RAGE-NF-kappaB signaling. Cell Death Dis. 2014;5(6): e1299. 24967961 10.1038/cddis.2014.258 PMC4611731

[171] Hartz AM, Zhong Y, Wolf A, LeVine H 3rd, Miller DS, Bauer B. Abeta40 reduces P-glycoprotein at the blood-brain barrier through the ubiquitin-proteasome pathway. J Neurosci. 2016;36(6):1930–41. 26865616 10.1523/JNEUROSCI.0350-15.2016 PMC4748076

[172] Cordone S, Annarumma L, Rossini PM, De Gennaro L. Sleep and beta-amyloid deposition in Alzheimer disease: insights on mechanisms and possible innovative treatments. Front Pharmacol. 2019;10:695. 31281257 10.3389/fphar.2019.00695 PMC6595048

[173] Yang J, Lunde LK, Nuntagij P, Oguchi T, Camassa LM, Nilsson LN, et al. Loss of astrocyte polarization in the tg-ArcSwe mouse model of Alzheimer’s disease. J Alzheimers Dis. 2011;27(4):711–22. 21891870 10.3233/JAD-2011-110725

[174] Mestre H, Hablitz LM, Xavier AL, Feng W, Zou W, Pu T, et al. Aquaporin-4-dependent glymphatic solute transport in the rodent brain. Elife. 2018;7:e40070. 30561329 10.7554/eLife.40070 PMC6307855

[175] Mazura AD, Ohler A, Storck SE, Kurtyka M, Scharfenberg F, Weggen S, et al. PCSK9 acts as a key regulator of abeta clearance across the blood-brain barrier. Cell Mol Life Sci. 2022;79(4):212. 35344086 10.1007/s00018-022-04237-x PMC8960591

[176] Cole SL, Vassar R. The Alzheimer’s disease beta-secretase enzyme, BACE1. Mol Neurodegener. 2007;2:22. 18005427 10.1186/1750-1326-2-22 PMC2211305

[177] Montagne A, Nation DA, Pa J, Sweeney MD, Toga AW, Zlokovic BV. Brain imaging of neurovascular dysfunction in Alzheimer’s disease. Acta Neuropathol. 2016;131(5):687–707. 27038189 10.1007/s00401-016-1570-0 PMC5283382

[178] Wierenga CE, Dev SI, Shin DD, Clark LR, Bangen KJ, Jak AJ, et al. Effect of mild cognitive impairment and APOE genotype on resting cerebral blood flow and its association with cognition. J Cereb Blood Flow Metab. 2012;32(8):1589–99. 22549621 10.1038/jcbfm.2012.58 PMC3421098

[179] Wirth M, Pichet Binette A, Brunecker P, Kobe T, Witte AV, Floel A. Divergent regional patterns of cerebral hypoperfusion and gray matter atrophy in mild cognitive impairment patients. J Cereb Blood Flow Metab. 2017;37(3):814–24. 27037094 10.1177/0271678X16641128 PMC5363461

[180] Schultz N, Brannstrom K, Byman E, Moussaud S, Nielsen HM, Netherlands Brain B, et al. Amyloid-beta 1–40 is associated with alterations in NG2+ pericyte population ex vivo and in vitro. Aging Cell. 2018;17(3): e12728. 29453790 10.1111/acel.12728 PMC5946076

[181] Abubaker AA, Vara D, Visconte C, Eggleston I, Torti M, Canobbio I, et al. Amyloid peptide beta1-42 induces integrin alphaiibbeta3 activation, platelet adhesion, and thrombus formation in a NADPH oxidase-dependent manner. Oxid Med Cell Longev. 2019;2019:1050476. 31007831 10.1155/2019/1050476 PMC6441506

[182] Park L, Zhou J, Koizumi K, Wang G, Anfray A, Ahn SJ, et al. tPA deficiency underlies neurovascular coupling dysfunction by amyloid-beta. J Neurosci. 2020;40(42):8160–73. 32928888 10.1523/JNEUROSCI.1140-20.2020 PMC7574658

[183] Albrecht D, Isenberg AL, Stradford J, Monreal T, Sagare A, Pachicano M, et al. Associations between vascular function and tau PET are associated with global cognition and amyloid. J Neurosci. 2020;40(44):8573–86. 33046556 10.1523/JNEUROSCI.1230-20.2020 PMC7605425

[184] Weller RO, Boche D, Nicoll JA. Microvasculature changes and cerebral amyloid angiopathy in Alzheimer’s disease and their potential impact on therapy. Acta Neuropathol. 2009;118(1):87–102. 19234858 10.1007/s00401-009-0498-z

[185] Saito S, Ihara M. Interaction between cerebrovascular disease and Alzheimer pathology. Curr Opin Psychiatry. 2016;29(2):168–73. 26779861 10.1097/YCO.0000000000000239

[186] Basun H, Bogdanovic N, Ingelsson M, Almkvist O, Naslund J, Axelman K, et al. Clinical and neuropathological features of the arctic APP gene mutation causing early-onset Alzheimer disease. Arch Neurol. 2008;65(4):499–505. 18413473 10.1001/archneur.65.4.499 PMC2723757

[187] Finch CE, Laping NJ, Morgan TE, Nichols NR, Pasinetti GM. TGF-beta 1 is an organizer of responses to neurodegeneration. J Cell Biochem. 1993;53(4):314–22. 8300749 10.1002/jcb.240530408

[188] Salminen A, Ojala J, Kaarniranta K, Haapasalo A, Hiltunen M, Soininen H. Astrocytes in the aging brain express characteristics of senescence-associated secretory phenotype. Eur J Neurosci. 2011;34(1):3–11. 21649759 10.1111/j.1460-9568.2011.07738.x

[189] Kato T, Sekine Y, Nozaki H, Uemura M, Ando S, Hirokawa S, et al. Excessive production of transforming growth factor beta1 causes mural cell depletion from cerebral small vessels. Front Aging Neurosci. 2020;12:151. 32581764 10.3389/fnagi.2020.00151 PMC7283554

[190] Boles Ponto LL, Magnotta VA, Moser DJ, Duff KM, Schultz SK. Global cerebral blood flow in relation to cognitive performance and reserve in subjects with mild memory deficits. Mol Imag Biol. 2006;8(6):363–72. 10.1007/s11307-006-0066-z 17048070

[191] den Abeelen AS, Lagro J, van Beek AH, Claassen JA. Impaired cerebral autoregulation and vasomotor reactivity in sporadic Alzheimer’s disease. Curr Alzheimer Res. 2014;11(1):11–7. 24251392 10.2174/1567205010666131119234845

[192] Yezhuvath US, Uh J, Cheng Y, Martin-Cook K, Weiner M, Diaz-Arrastia R, et al. Forebrain-dominant deficit in cerebrovascular reactivity in Alzheimer’s disease. Neurobiol Aging. 2012;33(1):75–82. 20359779 10.1016/j.neurobiolaging.2010.02.005 PMC2896562

[193] Okonkwo OC, Xu G, Oh JM, Dowling NM, Carlsson CM, Gallagher CL, et al. Cerebral blood flow is diminished in asymptomatic middle-aged adults with maternal history of Alzheimer’s disease. Cereb Cortex. 2014;24(4):978–88. 23236200 10.1093/cercor/bhs381 PMC3948496

[194] Rombouts SARB, Barkhof F, Veltman DJ, Machielsen WCM, Witter MP, Bierlaagh MA, et al. Functional MR imaging in Alzheimer’s disease during memory encoding. Am J Neuroradiol. 2000;21(10):1869–75. 11110539 PMC7974309

[195] Small SA, Perera GM, DeLaPaz R, Mayeux R, Stern Y. Differential regional dysfunction of the hippocampal formation among elderly with memory decline and Alzheimer’s disease. Ann Neurol. 1999;45(4):466–72. 10211471 10.1002/1531-8249(199904)45:4<466::AID-ANA8>3.0.CO;2-Q

[196] Hussong SA, Banh AQ, Van Skike CE, Dorigatti AO, Hernandez SF, Hart MJ, et al. Soluble pathogenic tau enters brain vascular endothelial cells and drives cellular senescence and brain microvascular dysfunction in a mouse model of tauopathy. Nat Commun. 2023;14(1):2367. 37185259 10.1038/s41467-023-37840-y PMC10126555

[197] Chakrabarty P, Jansen-West K, Beccard A, Ceballos-Diaz C, Levites Y, Verbeeck C, et al. Massive gliosis induced by interleukin-6 suppresses abeta deposition in vivo: evidence against inflammation as a driving force for amyloid deposition. FASEB J. 2010;24(2):548–59. 19825975 10.1096/fj.09-141754 PMC3083918

[198] Shaftel SS, Kyrkanides S, Olschowka JA, Miller JN, Johnson RE, O’Banion MK. Sustained hippocampal IL-1 beta overexpression mediates chronic neuroinflammation and ameliorates Alzheimer plaque pathology. J Clin Invest. 2007;117(6):1595–604. 17549256 10.1172/JCI31450 PMC1878531

[199] Krabbe G, Halle A, Matyash V, Rinnenthal JL, Eom GD, Bernhardt U, et al. Functional impairment of microglia coincides with Beta-amyloid deposition in mice with Alzheimer-like pathology. PLoS ONE. 2013;8(4): e60921. 23577177 10.1371/journal.pone.0060921 PMC3620049

[200] Michelucci A, Heurtaux T, Grandbarbe L, Morga E, Heuschling P. Characterization of the microglial phenotype under specific pro-inflammatory and anti-inflammatory conditions: effects of oligomeric and fibrillar amyloid-beta. J Neuroimmunol. 2009;210(1–2):3–12. 19269040 10.1016/j.jneuroim.2009.02.003

[201] Jay TR, Miller CM, Cheng PJ, Graham LC, Bemiller S, Broihier ML, et al. TREM2 deficiency eliminates TREM2+ inflammatory macrophages and ameliorates pathology in Alzheimer’s disease mouse models. J Exp Med. 2015;212(3):287–95. 25732305 10.1084/jem.20142322 PMC4354365

[202] Saresella M, Marventano I, Calabrese E, Piancone F, Rainone V, Gatti A, et al. A complex proinflammatory role for peripheral monocytes in Alzheimer’s disease. J Alzheimers Dis. 2014;38(2):403–13. 23979026 10.3233/JAD-131160

[203] Stamatovic SM, Shakui P, Keep RF, Moore BB, Kunkel SL, Van Rooijen N, et al. Monocyte chemoattractant protein-1 regulation of blood-brain barrier permeability. J Cereb Blood Flow Metab. 2005;25(5):593–606. 15689955 10.1038/sj.jcbfm.9600055

[204] Haddick PC, Larson JL, Rathore N, Bhangale TR, Phung QT, Srinivasan K, et al. A common variant of IL-6R is associated with elevated IL-6 pathway activity in Alzheimer’s disease brains. J Alzheimers Dis. 2017;56(3):1037–54. 28106546 10.3233/JAD-160524 PMC5667357

[205] Decourt B, Lahiri DK, Sabbagh MN. Targeting tumor necrosis factor alpha for Alzheimer’s disease. Curr Alzheimer Res. 2017;14(4):412–25. 27697064 10.2174/1567205013666160930110551 PMC5328927

[206] Forster C, Burek M, Romero IA, Weksler B, Couraud PO, Drenckhahn D. Differential effects of hydrocortisone and TNFalpha on tight junction proteins in an in vitro model of the human blood-brain barrier. J Physiol. 2008;586(7):1937–49. 18258663 10.1113/jphysiol.2007.146852 PMC2375735

[207] Hoshi Y, Uchida Y, Tachikawa M, Ohtsuki S, Terasaki T. Actin filament-associated protein 1 (AFAP-1) is a key mediator in inflammatory signaling-induced rapid attenuation of intrinsic P-gp function in human brain capillary endothelial cells. J Neurochem. 2017;141(2):247–62. 28112407 10.1111/jnc.13960

[208] Hartz AM, Bauer B, Fricker G, Miller DS. Rapid modulation of P-glycoprotein-mediated transport at the blood-brain barrier by tumor necrosis factor-alpha and lipopolysaccharide. Mol Pharmacol. 2006;69(2):462–70. 16278373 10.1124/mol.105.017954

[209] Ryu JK, McLarnon JG. A leaky blood-brain barrier, fibrinogen infiltration and microglial reactivity in inflamed Alzheimer’s disease brain. J Cell Mol Med. 2009;13(9A):2911–25. 18657226 10.1111/j.1582-4934.2008.00434.x PMC4498946

[210] Paul J, Strickland S, Melchor JP. Fibrin deposition accelerates neurovascular damage and neuroinflammation in mouse models of Alzheimer’s disease. J Exp Med. 2007;204(8):1999–2008. 17664291 10.1084/jem.20070304 PMC2118680

[211] Cortes-Canteli M, Zamolodchikov D, Ahn HJ, Strickland S, Norris EH. Fibrinogen and altered hemostasis in Alzheimer’s disease. J Alzheimers Dis. 2012;32(3):599–608. 22869464 10.3233/JAD-2012-120820 PMC3683985

[212] Zamolodchikov D, Strickland S. A possible new role for Aβ in vascular and inflammatory dysfunction in Alzheimer’s disease. Thromb Res. 2016;141:S59–61. 27207427 10.1016/S0049-3848(16)30367-X

[213] Qi J, Goralnick S, Kreutzer DL. Fibrin regulation of interleukin-8 gene expression in human vascular endothelial cells. Blood. 1997;90(9):3595–602. 9345043 10.1182/blood.V90.9.3595

[214] Yin X, Wright J, Wall T, Grammas P. Brain endothelial cells synthesize neurotoxic thrombin in Alzheimer’s disease. Am J Pathol. 2010;176(4):1600–6. 20150433 10.2353/ajpath.2010.090406 PMC2843451

[215] Grammas P. Neurovascular dysfunction, inflammation and endothelial activation: implications for the pathogenesis of Alzheimer’s disease. J Neuroinflammation. 2011;8:26. 21439035 10.1186/1742-2094-8-26 PMC3072921

[216] Petersen MA, Ryu JK, Akassoglou K. Fibrinogen in neurological diseases: mechanisms, imaging and therapeutics. Nat Rev Neurosci. 2018;19(5):283–301. 29618808 10.1038/nrn.2018.13 PMC6743980

[217] Verstraeten A, Theuns J, Van Broeckhoven C. Progress in unraveling the genetic etiology of Parkinson disease in a genomic era. Trend Genet. 2015;31(3):140–9. 10.1016/j.tig.2015.01.004 25703649

[218] Al-Bachari S, Naish JH, Parker GJM, Emsley HCA, Parkes LM. Blood-brain barrier leakage is increased in Parkinson’s disease. Front Physiol. 2020;11: 593026. 33414722 10.3389/fphys.2020.593026 PMC7784911

[219] Al-Bachari S, Naish JH, Parker GJM, Emsley HCA, Parkes LM. Blood-brain barrier leakage is increased in Parkinson’s disease. Front Physiol. 2020;11: 593026. 33414722 10.3389/fphys.2020.593026 PMC7784911

[220] Pajares M, Ana IR, Manda G, Bosca L, Cuadrado A. Inflammation in Parkinson’s disease: mechanisms and therapeutic implications. Cells. 2020. 10.3390/cells9071687. 32674367 10.3390/cells9071687 PMC7408280

[221] Chen X, Lan X, Roche I, Liu R, Geiger JD. Caffeine protects against MPTP-induced blood-brain barrier dysfunction in mouse striatum. J Neurochem. 2008;107(4):1147–57. 18808450 10.1111/j.1471-4159.2008.05697.x PMC3692355

[222] Wang W, Bodles-Brakhop AM, Barger SW. A role for P-glycoprotein in clearance of Alzheimer amyloid beta-peptide from the brain. Curr Alzheimer Res. 2016;13(6):615–20. 26971931 10.2174/1567205013666160314151012 PMC5102249

[223] Cirrito JR, Deane R, Fagan AM, Spinner ML, Parsadanian M, Finn MB, et al. P-glycoprotein deficiency at the blood-brain barrier increases amyloid-beta deposition in an Alzheimer disease mouse model. J Clin Invest. 2005;115(11):3285–90. 16239972 10.1172/JCI25247 PMC1257538

[224] Huang R, Gao Y, Duan Q, Zhang Q, He P, Chen J, et al. Endothelial LRP1-ICD accelerates cognition-associated alpha-synuclein pathology and neurodegeneration through PARP1 activation in a mouse model of Parkinson’s disease. Mol Neurobiol. 2023;60(2):979–1003. 36394710 10.1007/s12035-022-03119-4

[225] Wang Y, An R, Umanah GK, Park H, Nambiar K, Eacker SM, et al. A nuclease that mediates cell death induced by DNA damage and poly(ADP-ribose) polymerase-1. Science. 2016. 10.1126/science.aad6872. 27846469 10.1126/science.aad6872 PMC5134926

[226] Riffault B, Kourdougli N, Dumon C, Ferrand N, Buhler E, Schaller F, et al. Pro-brain-derived neurotrophic factor (proBDNF)-mediated p75NTR activation promotes depolarizing actions of GABA and increases susceptibility to epileptic seizures. Cereb Cortex. 2018;28(2):510–27. 27913431 10.1093/cercor/bhw385

[227] Dohgu S, Takata F, Matsumoto J, Kimura I, Yamauchi A, Kataoka Y. Monomeric alpha-synuclein induces blood-brain barrier dysfunction through activated brain pericytes releasing inflammatory mediators in vitro. Microvasc Res. 2019;124:61–6. 30885616 10.1016/j.mvr.2019.03.005

[228] Lan G, Wang P, Chan RB, Liu Z, Yu Z, Liu X, et al. Astrocytic VEGFA: an essential mediator in blood-brain-barrier disruption in Parkinson’s disease. Glia. 2022;70(2):337–53. 34713920 10.1002/glia.24109

[229] Pierzchlińska A, Kwaśniak-Butowska M, Sławek J, Droździk M, Białecka M. Arterial blood pressure variability and other vascular factors contribution to the cognitive decline in Parkinson’s disease. Molecules. 2021. 10.3390/molecules26061523. 33802165 10.3390/molecules26061523 PMC8001922

[230] Rite I, Machado A, Cano J, Venero JL. Blood-brain barrier disruption induces in vivo degeneration of nigral dopaminergic neurons. J Neurochem. 2007;101(6):1567–82. 17437543 10.1111/j.1471-4159.2007.04567.x

[231] Barcia C, Emborg ME, Hirsch EC, Herrero MT. Blood vessels and parkinsonism. Front Biosci. 2004;9:277–82. 14766365 10.2741/1145

[232] Guan J, Pavlovic D, Dalkie N, Waldvogel HJ, O’Carroll SJ, Green CR, et al. Vascular degeneration in Parkinson’s disease. Brain Pathol. 2013;23(2):154–64. 22897695 10.1111/j.1750-3639.2012.00628.x PMC8029297

[233] Nguyen B, Bix G, Yao Y. Basal lamina changes in neurodegenerative disorders. Mol Neurodegener. 2021;16(1):81. 34876200 10.1186/s13024-021-00502-y PMC8650282

[234] Ham JH, Yi H, Sunwoo MK, Hong JY, Sohn YH, Lee PH. Cerebral microbleeds in patients with Parkinson’s disease. J Neurol. 2014;261(8):1628–35. 24920492 10.1007/s00415-014-7403-y

[235] Biju KC, Shen Q, Hernandez ET, Mader MJ, Clark RA. Reduced cerebral blood flow in an α-synuclein transgenic mouse model of Parkinson’s disease. J Cerebr Blood F Met. 2020;40(12):2441–53. 10.1177/0271678X19895432 PMC7820695 31856640

[236] Paul G, Elabi OF. Microvascular changes in Parkinson’s disease- focus on the neurovascular unit. Front Aging Neurosci. 2022. 10.3389/fnagi.2022.853372. 35360216 10.3389/fnagi.2022.853372 PMC8960855

[237] Shimoji M, Pagan F, Healton EB, Mocchetti I. CXCR4 and CXCL12 expression is increased in the nigro-striatal system of Parkinson’s disease. Neurotox Res. 2009;16(3):318–28. 19551455 10.1007/s12640-009-9076-3

[238] Ding X, Zhang M, Gu R, Xu G, Wu H. Activated microglia induce the production of reactive oxygen species and promote apoptosis of co-cultured retinal microvascular pericytes. Graefes Arch Clin Exp Ophthalmol. 2017;255(4):777–88. 28074262 10.1007/s00417-016-3578-5

[239] Wu F, Liu L, Zhou H. Endothelial cell activation in central nervous system inflammation. J Leukoc Biol. 2017;101(5):1119–32. 28196850 10.1189/jlb.3RU0816-352RR

[240] Steinruecke M, Lonergan RM, Selvaraj BT, Chandran S, Diaz-Castro B, Stavrou M. Blood-CNS barrier dysfunction in amyotrophic lateral sclerosis: proposed mechanisms and clinical implications. J Cereb Blood Flow Metab. 2023;43(5):642–54. 36704819 10.1177/0271678X231153281 PMC10108188

[241] Garbuzova-Davis S, Hernandez-Ontiveros DG, Rodrigues MC, Haller E, Frisina-Deyo A, Mirtyl S, et al. Impaired blood-brain/spinal cord barrier in ALS patients. Brain Res. 2012;1469:114–28. 22750125 10.1016/j.brainres.2012.05.056

[242] Garbuzova-Davis S, Saporta S, Haller E, Kolomey I, Bennett SP, Potter H, et al. Evidence of compromised blood-spinal cord barrier in early and late symptomatic SOD1 mice modeling ALS. PLoS ONE. 2007;2(11): e1205. 18030339 10.1371/journal.pone.0001205 PMC2075163

[243] Kwan JY, Jeong SY, Van Gelderen P, Deng HX, Quezado MM, Danielian LE, et al. Iron accumulation in deep cortical layers accounts for MRI signal abnormalities in ALS: correlating 7 tesla MRI and pathology. PLoS ONE. 2012. 10.1371/journal.pone.0035241. 22529995 10.1371/journal.pone.0035241 PMC3328441

[244] Garbuzova-Davis S, Kurien C, Haller E, Eve DJ, Navarro S, Steiner G, et al. Human bone marrow endothelial progenitor cell transplantation into symptomatic ALS mice delays disease progression and increases motor neuron survival by repairing blood-spinal cord barrier. Sci Rep. 2019;9(1):5280. 30918315 10.1038/s41598-019-41747-4 PMC6437219

[245] Winkler EA, Sengillo JD, Sagare AP, Zhao Z, Ma Q, Zuniga E, et al. Blood-spinal cord barrier disruption contributes to early motor-neuron degeneration in ALS-model mice. Proc Natl Acad Sci U S A. 2014;111(11):E1035-1042. 24591593 10.1073/pnas.1401595111 PMC3964055

[246] van Vliet EA, Iyer AM, Mesarosova L, Colakoglu H, Anink JJ, van Tellingen O, et al. Expression and cellular distribution of P-glycoprotein and breast cancer resistance protein in amyotrophic lateral sclerosis patients. J Neuropathol Exp Neurol. 2020;79(3):266–76. 31999342 10.1093/jnen/nlz142 PMC7036662

[247] Bataveljic D, Nikolic L, Milosevic M, Todorovic N, Andjus PR. Changes in the astrocytic aquaporin-4 and inwardly rectifying potassium channel expression in the brain of the amyotrophic lateral sclerosis SOD1(G93A) rat model. Glia. 2012;60(12):1991–2003. 22987392 10.1002/glia.22414

[248] Wang L, Gutmann DH, Roos RP. Astrocyte loss of mutant SOD1 delays ALS disease onset and progression in G85R transgenic mice. Hum Mol Genet. 2011;20(2):286–93. 20962037 10.1093/hmg/ddq463

[249] Jo M, Lee S, Jeon YM, Kim S, Kwon Y, Kim HJ. The role of TDP-43 propagation in neurodegenerative diseases: integrating insights from clinical and experimental studies. Exp Mol Med. 2020;52(10):1652–62. 33051572 10.1038/s12276-020-00513-7 PMC8080625

[250] Lee S, Kim S, Kang HY, Lim HR, Kwon Y, Jo M, et al. The overexpression of TDP-43 in astrocytes causes neurodegeneration via a PTP1B-mediated inflammatory response. J Neuroinflammation. 2020;17(1):299. 33054766 10.1186/s12974-020-01963-6 PMC7556969

[251] Moreau C, Devos D, Brunaud-Danel V, Defebvre L, Perez T, Destee A, et al. Elevated IL-6 and TNF-alpha levels in patients with ALS: inflammation or hypoxia? Neurology. 2005;65(12):1958–60. 16380619 10.1212/01.wnl.0000188907.97339.76

[252] Zamudio F, Loon AR, Smeltzer S, Benyamine K, Navalpur Shanmugam NK, Stewart NJF, et al. TDP-43 mediated blood-brain barrier permeability and leukocyte infiltration promote neurodegeneration in a low-grade systemic inflammation mouse model. J Neuroinflammation. 2020;17(1):283. 32979923 10.1186/s12974-020-01952-9 PMC7519496

[253] Jara JH, Gautam M, Kocak N, Xie EF, Mao Q, Bigio EH, et al. MCP1-CCR2 and neuroinflammation in the ALS motor cortex with TDP-43 pathology. J Neuroinflammation. 2019;16(1):196. 31666087 10.1186/s12974-019-1589-y PMC6822373

[254] Garbuzova-Davis S, Ehrhart J, Sanberg PR, Borlongan CV. Potential role of humoral IL-6 cytokine in mediating pro-inflammatory endothelial cell response in amyotrophic lateral sclerosis. Int J Mol Sci. 2018. 10.3390/ijms19020423. 29385088 10.3390/ijms19020423 PMC5855645

[255] Yu W, He J, Cai X, Yu Z, Zou Z, Fan D. Neuroimmune crosstalk between the peripheral and the central immune system in amyotrophic lateral sclerosis. Front Aging Neurosci. 2022. 10.3389/fnagi.2022.890958. 35592701 10.3389/fnagi.2022.890958 PMC9110796

[256] Pieper C, Pieloch P, Galla HJ. Pericytes support neutrophil transmigration via interleukin-8 across a porcine co-culture model of the blood-brain barrier. Brain Res. 2013;1524:1–11. 23769734 10.1016/j.brainres.2013.05.047

[257] Ouali Alami N, Schurr C, Olde Heuvel F, Tang L, Li Q, Tasdogan A, et al. NF-kappaB activation in astrocytes drives a stage-specific beneficial neuroimmunological response in ALS. EMBO J. 2018. 10.15252/embj.201798697. 29875132 10.15252/embj.201798697 PMC6092622

[258] Jones MK, Nair A, Gupta M. Mast cells in neurodegenerative disease. Front Cell Neurosci. 2019;13:171. 31133804 10.3389/fncel.2019.00171 PMC6524694

[259] Dendrou CA, Fugger L, Friese MA. Immunopathology of multiple sclerosis. Nat Rev Immunol. 2015;15(9):545–58. 26250739 10.1038/nri3871

[260] McDonald WI, Compston A, Edan G, Goodkin D, Hartung HP, Lublin FD, et al. Recommended diagnostic criteria for multiple sclerosis: guidelines from the international panel on the diagnosis of multiple sclerosis. Ann Neurol. 2001;50(1):121–7. 11456302 10.1002/ana.1032

[261] Alvarez JI, Cayrol R, Prat A. Disruption of central nervous system barriers in multiple sclerosis. Bba-Mol Basis Dis. 2011;1812(2):252–64. 10.1016/j.bbadis.2010.06.017 20619340

[262] Gerwien H, Hermann S, Zhang XL, Korpos E, Song J, Kopka K, et al. Imaging matrix metalloproteinase activity in multiple sclerosis as a specific marker of leukocyte penetration of the blood-brain barrier. Sci Transl Med. 2016. 10.1126/scitranslmed.aaf8020. 27831901 10.1126/scitranslmed.aaf8020

[263] Barreiro O, Sanchez-Madrid F. Molecular basis of leukocyte-endothelium interactions during the inflammatory response. Rev Esp Cardiol. 2009;62(5):552–62. 19406069 10.1016/S0300-8932(09)71035-8

[264] Hernandez-Pedro NY, Espinosa-Ramirez G, de la Cruz VP, Pineda B, Sotelo J. Initial immunopathogenesis of multiple sclerosis: innate immune response. Clin Dev Immunol. 2013;2013: 413465. 24174969 10.1155/2013/413465 PMC3794540

[265] Prajeeth CK, Dittrich-Breiholz O, Talbot SR, Robert PA, Huehn J, Stangel M. IFN-gamma producing Th1 cells induce different transcriptional profiles in microglia and astrocytes. Front Cell Neurosci. 2018;12:352. 30364000 10.3389/fncel.2018.00352 PMC6191492

[266] Kunkl M, Frascolla S, Amormino C, Volpe E, Tuosto L. T helper cells: the modulators of inflammation in multiple sclerosis. Cells. 2020. 10.3390/cells9020482. 32093011 10.3390/cells9020482 PMC7072830

[267] Paintlia MK, Paintlia AS, Singh AK, Singh I. Synergistic activity of interleukin-17 and tumor necrosis factor-alpha enhances oxidative stress-mediated oligodendrocyte apoptosis. J Neurochem. 2011;116(4):508–21. 21143599 10.1111/j.1471-4159.2010.07136.x PMC3033460

[268] Balasa R, Barcutean L, Mosora O, Manu D. Reviewing the significance of blood-brain barrier disruption in multiple sclerosis pathology and treatment. Int J Mol Sci. 2021. 10.3390/ijms22168370. 34445097 10.3390/ijms22168370 PMC8395058

[269] Schreiner TG, Romanescu C, Popescu BO. The blood-brain barrier-a key player in multiple sclerosis disease mechanisms. Biomolecules. 2022. 10.3390/biom12040538. 35454127 10.3390/biom12040538 PMC9025898

[270] Correale J, Gaitan MI, Ysrraelit MC, Fiol MP. Progressive multiple sclerosis: from pathogenic mechanisms to treatment. Brain. 2017;140(3):527–46. 27794524 10.1093/brain/aww258

[271] D’Haeseleer M, Cambron M, Vanopdenbosch L, De Keyser J. Vascular aspects of multiple sclerosis. Lancet Neurol. 2011;10(7):657–66. 21683931 10.1016/S1474-4422(11)70105-3

[272] Monti L, Morbidelli L, Rossi A. Impaired cerebral perfusion in multiple sclerosis: relevance of endothelial factors. Biomark Insights. 2018. 10.1177/1177271918774800. 29795976 10.1177/1177271918774800 PMC5960845

[273] De Keyser J, Wilczak N, Leta R, Streetland C. Astrocytes in multiple sclerosis lack beta-2 adrenergic receptors. Neurology. 1999;53(8):1628–33. 10563603 10.1212/WNL.53.8.1628

[274] D’haeseleer M, Hostenbach S, Peeters I, El Sankari S, Nagels G, De Keyser J, et al. Cerebral hypoperfusion: a new pathophysiologic concept in multiple sclerosis? J Cerebr Blood F Met. 2015;35(9):1406–10. 10.1038/jcbfm.2015.131 PMC4640326 26104292

[275] Ouellette J, Lacoste B. From neurodevelopmental to neurodegenerative disorders: the vascular continuum. Front Aging Neurosci. 2021. 10.3389/fnagi.2021.749026. 34744690 10.3389/fnagi.2021.749026 PMC8570842

[276] Marshall O, Lu HZ, Brisset JC, Xu F, Liu PY, Herbert J, et al. Impaired cerebrovascular reactivity in multiple sclerosis. Jama Neurol. 2014;71(10):1275–81. 25133874 10.1001/jamaneurol.2014.1668 PMC4376108

[277] Wakefield AJ, More LJ, Difford J, Mclaughlin JE. Immunohistochemical study of vascular injury in acute multiple-sclerosis. J Clin Pathol. 1994;47(2):129–33. 8132826 10.1136/jcp.47.2.129 PMC501826

[278] Lyons SA, Kettenmann H. Oligodendrocytes and microglia are selectively vulnerable to combined hypoxia and hypoglycemia injury in vitro. J Cereb Blood Flow Metab. 1998;18(5):521–30. 9591844 10.1097/00004647-199805000-00007

[279] Juhler M, Laursen H, Barry DI. The distribution of immunoglobulins and albumin in the central nervous system in acute experimental allergic encephalomyelitis. Acta Neurol Scand. 2009;73(2):119–24. 10.1111/j.1600-0404.1986.tb03251.x 3518328

[280] Vega-Zelaya L, Ortega GJ, Sola RG, Pastor J. Plasma albumin induces cytosolic calcium oscilations and DNA synthesis in human cultured astrocytes. Biomed Res Int. 2014;2014:1–10. 10.1155/2014/539140 PMC4054939 24967376

[281] van Vliet EA, Aronica E, Gorter JA. Blood-brain barrier dysfunction, seizures and epilepsy. Semin Cell Dev Biol. 2015;38:26–34. 25444846 10.1016/j.semcdb.2014.10.003

[282] Tabernero A, Granda B, Medina A, Sanchez-Abarca LI, Lavado E, Medina JM. Albumin promotes neuronal survival by increasing the synthesis and release of glutamate. J Neurochem. 2002;81(4):881–91. 12065647 10.1046/j.1471-4159.2002.00843.x

[283] Andravizou A, Dardiotis E, Artemiadis A, Sokratous M, Siokas V, Tsouris Z, et al. Brain atrophy in multiple sclerosis: mechanisms, clinical relevance and treatment options. Auto Immun Highlights. 2019;10(1):7. 32257063 10.1186/s13317-019-0117-5 PMC7065319

[284] Choi BY, Jung JW, Suh SW. The emerging role of zinc in the pathogenesis of multiple sclerosis. Int J Mol Sci. 2017. 10.3390/ijms18102070. 28956834 10.3390/ijms18102070 PMC5666752

[285] Griffin JH, Zlokovic BV, Mosnier LO. Activated protein C: biased for translation. Blood. 2015;125(19):2898–907. 25824691 10.1182/blood-2015-02-355974 PMC4424414

[286] Cheng T, Petraglia AL, Li Z, Thiyagarajan M, Zhong Z, Wu Z, et al. Activated protein C inhibits tissue plasminogen activator-induced brain hemorrhage. Nat Med. 2006;12(11):1278–85. 17072311 10.1038/nm1498

[287] Han MH, Hwang SI, Roy DB, Lundgren DH, Price JV, Ousman SS, et al. Proteomic analysis of active multiple sclerosis lesions reveals therapeutic targets. Nature. 2008;451(7182):1076–81. 18278032 10.1038/nature06559

[288] Lazic D, Sagare AP, Nikolakopoulou AM, Griffin JH, Vassar R, Zlokovic BV. 3K3A-activated protein C blocks amyloidogenic BACE1 pathway and improves functional outcome in mice. J Exp Med. 2019;216(2):279–93. 30647119 10.1084/jem.20181035 PMC6363429

[289] Zhong Z, Ilieva H, Hallagan L, Bell R, Singh I, Paquette N, et al. Activated protein C therapy slows ALS-like disease in mice by transcriptionally inhibiting SOD1 in motor neurons and microglia cells. J Clin Invest. 2009;119(11):3437–49. 19841542 10.1172/JCI38476 PMC2769191

[290] Griffin JH, Zlokovic BV, Mosnier LO. Activated protein C, protease activated receptor 1, and neuroprotection. Blood. 2018;132(2):159–69. 29866816 10.1182/blood-2018-02-769026 PMC6043978

[291] Bell RD, Winkler EA, Singh I, Sagare AP, Deane R, Wu Z, et al. Apolipoprotein E controls cerebrovascular integrity via cyclophilin A. Nature. 2012;485(7399):512–6. 22622580 10.1038/nature11087 PMC4047116

[292] Fei Yx, Zhu Jp, Zhao B, Yin Qy, Fang Wr, Li Ym. XQ-1H regulates Wnt/GSK3β/β-catenin pathway and ameliorates the integrity of blood brain barrier in mice with acute ischemic stroke. Brain Res Bull. 2020;164:269–88. 32916221 10.1016/j.brainresbull.2020.08.032

[293] Xu L, Nirwane A, Xu T, Kang M, Devasani K, Yao Y. Fibroblasts repair blood-brain barrier damage and hemorrhagic brain injury via TIMP2. Cell Rep. 2022;41(8): 111709. 36417884 10.1016/j.celrep.2022.111709 PMC9769996

[294] Braniste V, Al-Asmakh M, Kowal C, Anuar F, Abbaspour A, Toth M, et al. The gut microbiota influences blood-brain barrier permeability in mice. Sci Transl Med. 2014;6(263): 263ra158. 25411471 10.1126/scitranslmed.3009759 PMC4396848

[295] Geng J, Wang L, Qu M, Song Y, Lin X, Chen Y, et al. Endothelial progenitor cells transplantation attenuated blood-brain barrier damage after ischemia in diabetic mice via HIF-1alpha. Stem Cell Res Ther. 2017;8(1):163. 28697748 10.1186/s13287-017-0605-3 PMC5505148

[296] Leeper NJ, Hunter AL, Cooke JP. Stem cell therapy for vascular regeneration: adult, embryonic, and induced pluripotent stem cells. Circulation. 2010;122(5):517–26. 20679581 10.1161/CIRCULATIONAHA.109.881441 PMC2920605

[297] Nikolakopoulou AM, Wang Y, Ma Q, Sagare AP, Montagne A, Huuskonen MT, et al. Endothelial LRP1 protects against neurodegeneration by blocking cyclophilin A. J Exp Med. 2021. 10.1084/jem.20202207. 33533918 10.1084/jem.20202207 PMC7863706

[298] Sui YT, Bullock KM, Erickson MA, Zhang J, Banks WA. Alpha synuclein is transported into and out of the brain by the blood-brain barrier. Peptides. 2014;62:197–202. 25278492 10.1016/j.peptides.2014.09.018 PMC4378645

[299] Brinton RD, Swanson HM, Irwin RW. P4–026: allopregnanolone promotes cholesterol and amyloid-beta clearance mechanisms: assessment of a regenerative therapeutic for Alzheimer’s disease. Alzheimer Dement. 2016;12:1024–5. 10.1016/j.jalz.2016.06.2115

[300] Deane R, Du Yan S, Submamaryan RK, LaRue B, Jovanovic S, Hogg E, et al. RAGE mediates amyloid-beta peptide transport across the blood-brain barrier and accumulation in brain. Nat Med. 2003;9(7):907–13. 12808450 10.1038/nm890

[301] Huang Y, Chen S, Luo Y, Han Z. Crosstalk between inflammation and the BBB in stroke. Curr Neuropharmacol. 2020;18(12):1227–36. 32562523 10.2174/1570159X18666200620230321 PMC7770647

[302] Blecharz KG, Haghikia A, Stasiolek M, Kruse N, Drenckhahn D, Gold R, et al. Glucocorticoid effects on endothelial barrier function in the murine brain endothelial cell line cEND incubated with sera from patients with multiple sclerosis. Mult Scler. 2010;16(3):293–302. 20203147 10.1177/1352458509358189

[303] Dong X, Gao J, Zhang CY, Hayworth C, Frank M, Wang Z. Neutrophil membrane-derived nanovesicles alleviate inflammation to protect mouse brain injury from ischemic stroke. ACS Nano. 2019;13(2):1272–83. 30673266 10.1021/acsnano.8b06572 PMC6424134

[304] Chao C-C, Gutiérrez-Vázquez C, Rothhammer V, Mayo L, Wheeler MA, Tjon EC, et al. Metabolic control of astrocyte pathogenic activity via cPLA2-MAVS. Cell. 2019;179(7):1483-1498.e1422. 31813625 10.1016/j.cell.2019.11.016 PMC6936326

[305] Koistinaho M, Malm TM, Kettunen MI, Goldsteins G, Starckx S, Kauppinen RA, et al. Minocycline protects against permanent cerebral ischemia in wild type but not in matrix metalloprotease-9-deficient mice. J Cereb Blood Flow Metab. 2005;25(4):460–7. 15674236 10.1038/sj.jcbfm.9600040

[306] Salvador E, Shityakov S, Förster C. Glucocorticoids and endothelial cell barrier function. Cell Tissue Res. 2014;355(3):597–605. 24352805 10.1007/s00441-013-1762-z PMC3972429

[307] Li J, Zheng M, Shimoni O, Banks WA, Bush AI, Gamble JR, et al. Development of novel therapeutics targeting the blood-brain barrier: from barrier to carrier. Adv Sci. 2021. 10.1002/advs.202101090. 10.1002/advs.202101090 PMC8373165 34085418

[308] Jarrahi A, Braun M, Ahluwalia M, Gupta RV, Wilson M, Munie S, et al. Revisiting traumatic brain injury: from molecular mechanisms to therapeutic interventions. Biomedicines. 2020. 10.3390/biomedicines8100389. 33003373 10.3390/biomedicines8100389 PMC7601301

[309] Carrano A, Hoozemans JJM, van der Vies SM, van Horssen J, de Vries HE, Rozemuller AJM. Neuroinflammation and blood-brain barrier changes in capillary amyloid angiopathy. Neurodegener Dis. 2012;10(1–4):329–31. 22301467 10.1159/000334916

[310] Nehra G, Bauer B, Hartz AMS. Blood-brain barrier leakage in Alzheimer’s disease: from discovery to clinical relevance. Pharmacol Therapeut. 2022. 10.1016/j.pharmthera.2022.108119. 10.1016/j.pharmthera.2022.108119 PMC9107516 35108575

